# Brain developmental and cortical connectivity changes in transgenic monkeys carrying the human-specific duplicated gene *SRGAP2C*

**DOI:** 10.1093/nsr/nwad281

**Published:** 2023-11-03

**Authors:** Xiaoyu Meng, Qiang Lin, Xuerui Zeng, Jin Jiang, Min Li, Xin Luo, Kaimin Chen, Haixu Wu, Yan Hu, Cirong Liu, Bing Su

**Affiliations:** State Key Laboratory of Genetic Resources and Evolution, Kunming Institute of Zoology, Chinese Academy of Sciences, Kunming 650223, China; National Resource Center for Non-Human Primates, Kunming Primate Research Center, and National Research Facility for Phenotypic and Genetic Analysis of Model Animals (Primate Facility), Kunming Institute of Zoology, Chinese Academy of Sciences, Kunming 650107, China; Kunming College of Life Science, University of Chinese Academy of Sciences, Beijing 100101, China; State Key Laboratory of Genetic Resources and Evolution, Kunming Institute of Zoology, Chinese Academy of Sciences, Kunming 650223, China; State Key Laboratory of Genetic Resources and Evolution, Kunming Institute of Zoology, Chinese Academy of Sciences, Kunming 650223, China; National Resource Center for Non-Human Primates, Kunming Primate Research Center, and National Research Facility for Phenotypic and Genetic Analysis of Model Animals (Primate Facility), Kunming Institute of Zoology, Chinese Academy of Sciences, Kunming 650107, China; Kunming College of Life Science, University of Chinese Academy of Sciences, Beijing 100101, China; State Key Laboratory of Genetic Resources and Evolution, Kunming Institute of Zoology, Chinese Academy of Sciences, Kunming 650223, China; State Key Laboratory of Genetic Resources and Evolution, Kunming Institute of Zoology, Chinese Academy of Sciences, Kunming 650223, China; State Key Laboratory of Genetic Resources and Evolution, Kunming Institute of Zoology, Chinese Academy of Sciences, Kunming 650223, China; National Resource Center for Non-Human Primates, Kunming Primate Research Center, and National Research Facility for Phenotypic and Genetic Analysis of Model Animals (Primate Facility), Kunming Institute of Zoology, Chinese Academy of Sciences, Kunming 650107, China; State Key Laboratory of Genetic Resources and Evolution, Kunming Institute of Zoology, Chinese Academy of Sciences, Kunming 650223, China; National Resource Center for Non-Human Primates, Kunming Primate Research Center, and National Research Facility for Phenotypic and Genetic Analysis of Model Animals (Primate Facility), Kunming Institute of Zoology, Chinese Academy of Sciences, Kunming 650107, China; Kunming College of Life Science, University of Chinese Academy of Sciences, Beijing 100101, China; State Key Laboratory of Genetic Resources and Evolution, Kunming Institute of Zoology, Chinese Academy of Sciences, Kunming 650223, China; National Resource Center for Non-Human Primates, Kunming Primate Research Center, and National Research Facility for Phenotypic and Genetic Analysis of Model Animals (Primate Facility), Kunming Institute of Zoology, Chinese Academy of Sciences, Kunming 650107, China; Kunming College of Life Science, University of Chinese Academy of Sciences, Beijing 100101, China; State Key Laboratory of Genetic Resources and Evolution, Kunming Institute of Zoology, Chinese Academy of Sciences, Kunming 650223, China; Center for Excellence in Brain Science and Intelligence Technology, Institute of Neuroscience, CAS Key Laboratory of Primate Neurobiology, Chinese Academy of Sciences, Shanghai 200031, China; State Key Laboratory of Genetic Resources and Evolution, Kunming Institute of Zoology, Chinese Academy of Sciences, Kunming 650223, China; National Resource Center for Non-Human Primates, Kunming Primate Research Center, and National Research Facility for Phenotypic and Genetic Analysis of Model Animals (Primate Facility), Kunming Institute of Zoology, Chinese Academy of Sciences, Kunming 650107, China; Center for Excellence in Animal Evolution and Genetics, Chinese Academy of Sciences, Kunming 650223, China

**Keywords:** *SRGAP2C*, transgenic monkey, brain evolution, brain development, neurogenesis, neoteny

## Abstract

Human-specific duplicated genes contributed to phenotypic innovations during the origin of our own species, such as an enlarged brain and highly developed cognitive abilities. While prior studies on transgenic mice carrying the human-specific *SRGAP2C* gene have shown enhanced brain connectivity, the relevance to humans remains unclear due to the significant evolutionary gap between humans and rodents. In this study, to investigate the phenotypic outcome and underlying genetic mechanism of *SRGAP2C*, we generated transgenic cynomolgus macaques (*Macaca fascicularis*) carrying the human-specific *SRGAP2C* gene. Longitudinal MRI imaging revealed delayed brain development with region-specific volume changes, accompanied by altered myelination levels in the temporal and occipital regions. On a cellular level, the transgenic monkeys exhibited increased deep-layer neurons during fetal neurogenesis and delayed synaptic maturation in adolescence. Moreover, transcriptome analysis detected neotenic expression in molecular pathways related to neuron ensheathment, synaptic connections, extracellular matrix and energy metabolism. Cognitively, the transgenic monkeys demonstrated improved motor planning and execution skills. Together, our findings provide new insights into the mechanisms by which the newly evolved gene shapes the unique development and circuitry of the human brain.

## INTRODUCTION

Recent advancements in comparative genomics have revealed several key genetic mechanisms underlying the origin of the human brain, including the rapid evolution of protein-coding genes [1], human-specific gene duplications or deletions [2], and human-specific regulatory changes in the non-coding regions [3,4]. Given gene duplication is the major source of the newly originated genes in the genome during evolution [5,6], many human-specific duplicated genes (HSDGs) have been reported, and remarkably, several HSDGs (such as *ARHGAP11B* and *NOTCH2NL*) have been shown to promote basal progenitor amplification, cortical neurogenesis and cortex folding, highlighting the importance of HSDGs in shaping the molecular programs of human corticogenesis [7–11].

SLIT-ROBO Rho-GTPase-activating protein 2 (*SRGAP2A*) is a conserved gene in all mammals [12], and during evolution, a series of duplication events of this gene occurred in the hominin (the group consisting of modern humans, extinct human species and all our immediate ancestors) lineage ∼3.4–1.0 million years ago [5,13]. The origin of the human-specific copy (*SRGAP2C*) occurred ∼2.4 million years ago, a critical period for *Homo sapiens* speciation. *SRGAP2C* is currently fixed in modern humans, and it encodes a truncated version of *SRGAP2A* with a loss of almost the entire F-BARx domain. At the same time, *SRGAP2C* also accumulated many amino acid changes compared to the ancestral copy, the signature of Darwinian positive selection in shaping the novel function of this human-specific gene copy [13,14].

Due to the sequence similarity of *SRGAP2C* to its ancestral copy *SRGAP2A*, the *SRGAP2C* protein functions as an antagonist and inhibits all functions of the ancestral protein [13,14]. The expression of *SRGAP2C* in the mouse cortical region mimics the *SRGAP2A* knock-down phenotypes, leading to an increase in density of the long-neck thin-head dendritic spines [14]. The humanized mice carrying *SRGAP2C* exhibit an elevated number of excitatory synapses received by layer 2/3 pyramidal neurons (PNs), specifically stemming from enhanced local and long-range cortico–cortical connections. These findings demonstrate how *SRGAP2C* modifies circuit connectivity and facilitates behavioral performance associated with the sensory cortex [15].

Considering the deep divergence of the brain between humans and mice in terms of size and complexity, current knowledge on the functional roles of *SRGAP2C* needs to be further tested and updated using experimental animals that are relatively close to the human. Non-human primates (Old World monkeys such as macaques and New World monkeys such as common marmosets) serve this purpose, as demonstrated in the studies of *MCPH1* (a brain-development-related gene containing multiple human-specific amino acid changes) [16] and *ARHGAP11B* [8]. In this study, we generated transgenic cynomolgus monkeys (*Macaca fascicularis*) carrying the human-specific *SRGAP2C*. Utilizing combined approaches, including MRI brain imaging, transcriptome analysis, neuronal dye microinjection and behavioral/cognitive tests, we intended to explore the genetic role of *SRGAP2C* underlying human brain evolution, especially in the formation of the neural networks of the brain.

## RESULTS

### Generation of transgenic monkeys carrying human-specific *SRGAP2C* copies

**Figure 1.**
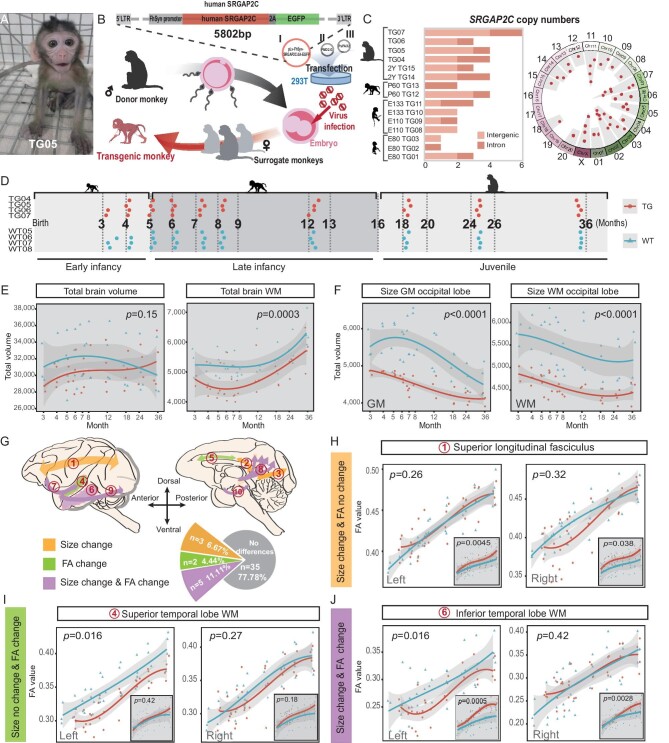
Generation of *SRGAP2C* TG monkeys and longitudinal brain imaging tracking. (A) Image of the TG monkey TG05 in early infancy. (B) Schematic illustration of the generation of TG monkeys, with plasmids (I–III): I, pLv-FhSyn-SRGAP2C-2A-EGFP; II, PMD2.G; III, PsPAX2. Upper panel, structure of the lentiviral vector with an inserted *SRGAP2C* gene copy next to the EGFP gene copy. EGFP, enhanced green fluorescence protein; LTR, long terminal repeat. (C) Validation of *SRGAP2C* copy number in the TG monkeys: left panel, variation in copy number among the TG monkeys; right panel, random distribution of the integrated *SRGAP2C* gene copies across chromosomes. (D) Timeline for longitudinal MRI data collection. (E) Brain volume changes: left, total brain volume; right, total WM volume, with age represented on a logarithmically scaled horizontal axis. (F) Volume changes of occipital lobe: left, gray matter volume; right, WM volume. (G) Overview of WM maturation patterns illustrated schematically. The pie chart displays the proportions of tracts with different maturation patterns. (H) Uncinate fasciculus depicted as a representative tract with size (embedded figure), but no FA, changes; left, left-hemisphere; right, right-hemisphere. (I) Superior temporal lobe WM illustrated as a representative tract with FA, but no size (embedded figure) changes. (J) Inferior temporal lobe WM depicted as a representative tract with both size (embedded figure) and FA differences. In (E–J), gray and white backgrounds correspond to T1-based size and FA-value, respectively. Group effect significance assessed via linear mixed model (LMM), with *P* < 0.05 considered significant.

High titer (>$1 \times {10}^{10}$ infection particles per ml) lentivirus carrying synapsin-promoter-driven human *SRGAP2C* copy and enhanced green fluorescence protein (EGFP) was injected into the perivitelline space of the mature oocytes of cynomolgus macaques after *in vitro* fertilization (IVF) (Fig. 1B). Embryos were then transferred into 42 surrogate monkeys, and 11 surrogates (11 out of 42, 26%) became pregnant (Fig. 1A and B). In addition, to obtain transgenic monkeys with relatively stable *SRGAP2C* copy numbers, we performed an intracytoplasmic sperm injection (ICSI) using sperm obtained from TG04 after he reached sexual maturity (Supplementary Fig. S1A). In total, we obtained 15 transgenic (TG) monkeys, including 8 live births (3 males and 5 females) and 8 fetal monkeys that were sampled during the gestation stages of E80 (3 fetal monkeys from a triplet pregnancy), E108–110 (two fetal monkeys) and E133 (two fetal monkeys) (Table 1). Among the eight live births, four were sacrificed for brain tissue sampling (two monkeys at the age of 60–70 days after birth, and two monkeys at the age of 783 days) (Table 1). All monkeys tested positive for the *SRGAP2C* transgene, as determined by polymerase chain reaction (PCR) (Supplementary Fig. S1).

**Table 1.** Information on the generated TG monkeys.

Monkey ID	Generation	Sex	Status	*SRGAP2C* copy number
TG01	F0	F	Sampled at 80 days (embryonic stage)	3
TG02	F0	F	Sampled at 80 days (embryonic stage)	1
TG03	F0	M	Sampled at 80 days (embryonic stage)	1
TG04	F0	M	Live, birth at 21/11/2015	4
TG05	F0	F	Live, birth at 9/12/2015	4
TG06	F0	F	Live, birth at 21/12/2015	4
TG07	F0	F	Live, birth at 21/11/2015	6
TG08	F1	M	Sampled at 110 days (embryonic stage)	4
TG09	F1	F	Sampled at 108 days (embryonic stage)	2
TG10	F1	F	Sampled at 135 days (embryonic stage)	4
TG11	F1	F	Sampled at 135 days (embryonic stage)	3
TG12	F0	F	Live, sampled 60 days after birth	4
TG13	F0	M	Live, sampled 70 days after birth	2
TG14	F0	F	Live, sampled 783 days after birth	4
TG15	F0	M	Live, sampled 783 days after birth	3

We employed the DNA walking method to determine the integrated genomic locations and copy numbers of the transgene [8]. As expected, the human *SRGAP2C* copies were randomly integrated into the monkey genome, ranging from one to six copies, and all transgenes were located in the genomic loci distant from known coding exons, and thus unlikely to interfere with the endogenous genes (Fig. 1C).

For comparison, we recruited 17 developmental-stage-matched wild-type (WT) monkeys (Supplementary Table S1), including 4 live monkeys separated from their biological mothers 3–7 days after birth and raised by humans in order to match the same conditions as the TG monkeys.

### Longitudinal MRI and DTI tracking reveal region-specific maturation patterns

*SRGAP2C*, a gene duplication specific to humans and essential for brain development, may lead to a changed brain maturation pattern in TG monkeys. To longitudinally track the developmental changes, we collected magnetic resonance imaging (MRI) and diffusion tensor imaging (DTI) data from four TG monkeys (TG04 to TG07) and four WT monkeys (WT05 to WT08), spanning from early infancy (3 months) to the juvenile stage (36 months). Due to the rapid development of the brain in its early stages, six scans were performed at 1-month intervals prior to the age of 9 months, followed by four additional scans at 12, 18, 24 and 36 months, respectively (Fig. 1D). Utilizing T1-weighted images, we assessed developmental changes in total brain volume, 13 cortical areas and 5 subcortical regions, according to the tUNC atlas [17].

At 3 months of age, the TG monkeys exhibited significantly smaller brain sizes than the WT monkeys, suggesting a slower brain development (Fig. 1E). The smaller brain size in early infancy also correlated with reduced body weight in the TG monkeys, although this association gradually diminished over time (Supplementary Fig. S2K), as evident from the curve of normalized brain volume. The WT monkeys attained peaks of total brain and gray matter volumes during late infancy and early juvenile stages, respectively. In contrast, these volumes in the TG monkeys continued to grow beyond 36 months (Fig. 1E and Supplementary Fig. S2B–F). Although both TG and WT monkeys exhibited comparable rates of total white matter (WM) volume growth, the absolute volume in the TG monkeys remained consistently lower than the WT controls (Supplementary Fig. S2B). These results revealed a pattern of delayed and prolonged brain development in TG monkeys relative to WT controls.

Beyond the general delay in development, the TG monkeys displayed noteworthy region-specific changes in brain maturation. In terms of gray matter volume, we observed three distinct patterns when comparing with the WT monkeys: (i) a consistently lower volume in the occipital lobe, along with a reduced proportion relative to total brain volume (Fig. 1F and Supplementary Fig. S3F); (ii) a consistently higher volume in the somatosensory cortex (Supplementary Fig. S2N); and (iii) a prolonged maturation in the primary motor cortex and temporal gyrus (Supplementary Fig. S2O and P). Unlike the varied patterns in gray matter, most WM regions followed similar growth in both TG and WT monkeys (Supplementary Fig. S2D–J). The only exception was the WM in the prefrontal lobe, which was found to be smaller in the TG monkeys (Supplementary Fig. S2C, LMM, group effect, right panel, *P* = 0.015). Specifically, the occipital lobe, the only region with a decreasing trend, revealed differences, with the TG monkeys exhibiting lower volume than the WT monkeys across all developmental stages (Fig. 1F, LMM, group effect, left panel, *P* < 0.0001; right panel, *P* < 0.0001). We also analyzed regional volume changes using another more fine-grained atlas of the macaque brain [18], and reaffirmed these regional differences (Supplementary Fig. S2L and M). To rule out gene dosage effects, we analyzed the correlation between the number of integrated *SRGAP2C* copies and various brain structural measurements, and no significant correlations were found, suggesting negligible effects of gene dosage (Supplementary Fig. S5).

While the WM volume in most brain lobes followed similar developmental trends, various WM tracts traversing different lobes may be selectively influenced by the *SRGAP2C* gene. Through tract-based DTI analysis, we found that an increase in WM volume did not uniformly lead to accelerated myelination, as indicated by the inconsistent fractional anisotropy (FA) and mean diffusivity (MD) values in these tracts (Fig. 1G). Specifically, we observed: (i) tracts undergoing volume changes without corresponding changes in FA (Fig. 1H, Supplementary Fig. S4A–C); (ii) tracts exhibiting FA changes but no volume difference (Fig. 1I and Supplementary Fig. S4D); and (iii) tracts demonstrating concurrent significant changes in both volume and FA (Fig. 1J and Supplementary Fig. S4F–H). In addition, the differences in tract size or FA values were seen in specific brain regions, such as the temporal lobe (Fig. 1G). Interestingly, we identified lateralized (left-hemispheric) developmental changes in WM tracts associated with the temporal lobe, including superior and inferior temporal gyrus WM (Fig. 1I and J). In particular, the uncinate fasciculus tract, which contributes to vocal and social communication, also displayed left-hemispheric lateralization in the TG monkeys. This lateralization mirrors the patterns reported in the human brain, which are believed to be associated with the evolution of language (Fig. 1H) [19,20]. These findings suggest that the introduction of *SRGAP2C* may have altered the myelination levels in the temporal and occipital regions, potentially affecting areas of the brain in the TG monkeys that are linked to visual and social recognition.

Summarizing our findings, the introduction of the human-specific *SRGAP2C* gene resulted in the generally delayed developmental trajectory of the monkey brain. Notably, this delay was not uniform across all brain regions. Instead, it was highly specific to areas associated with advanced cognitive functions in primates. These region-specific changes hint at the gene's potential influence on primate brain evolution, particularly in areas related to complex cognitive abilities.

### Increased neuron abundance in the visual cortex of TG monkeys

Our MRI data indicated that *SRGAP2C* may preferentially influence the occipital lobe, which houses the visual cortex. The visual cortex processes visual information and is the largest area in the brain dedicated to processing sensory information. It is present in humans and closely related species and its role in brain evolution has been proposed.

**Figure 2.**
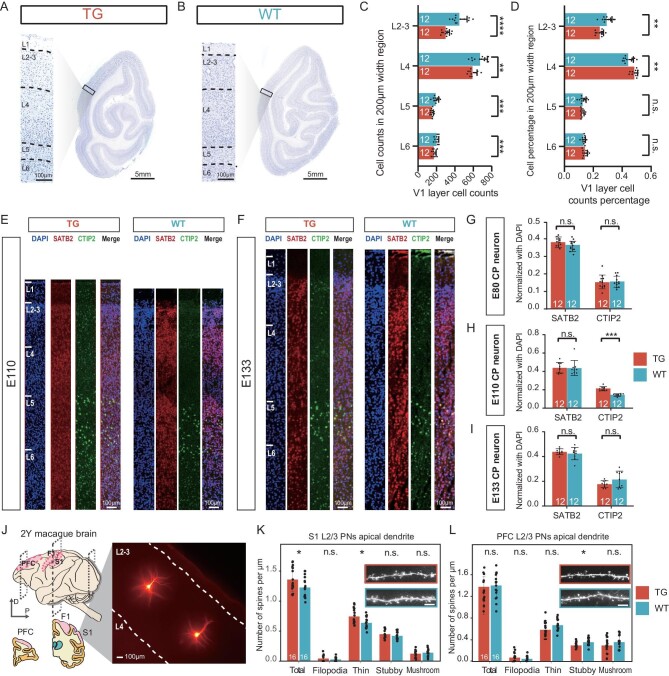
Increased cortical neuron production and delayed spinogenesis in TG monkeys. (A–B) Nissl staining of the V1 cortex in the 2-year-old TG and WT monkeys. The zoomed-in images are presented for both the TG and the WT monkeys (scale bar, 100 μm, 5 mm). (C) Quantification of cell numbers of the V1 cortex in a 200-μm-wide region. The data include two monkeys from each group (TG or WT), and six regions per monkey (unpaired Student's t test, **P < 0.01, ***P < 0.001, ****P < 0.0001). (D) Quantification of cell abundance of the V1 cortex in a 200-μm-wide region. The data include two monkeys from each group (TG or WT), and six regions per monkey (unpaired Student's t test, n.s. *P* > 0.05, **P < 0.01). (E–F) Immunofluorescent staining of SATB2 and CTIP2, combined with DAPI was performed for the TG and WT neocortex at the position where cortical thickness was measured. (E) E110, (F) E133 (scale bar, 100 μm). (G–I) Quantification of the SATB2^+^ neurons and the CTIP2^+^ neurons normalized by DAPI^+^ at (G) E80, (H) E110 and (I) E133 in a 200-μm-wide region of the TG and WT neocortex, including 4–6 sections per group (unpaired Student's t test, ***P < 0.001). (J) Schematic diagram showing the brain lateral view of the sampled 2-year-old macaques. The dashed-line boxes indicate the analyzed coronal section. The image of Alexa-Fluor-568-filled neurons in the monkey brain slice is shown (scale bar, 100 μm). (K–L) Summary data of the density of apical dendritic spines and subtypes of filopodia (immature), thin (immature), stubby (mature) and mushroom (mature) of the L2/3 pyramidal neurons in the (K) S1 and (L) PFC. The pictures of the analyzed dendrites are shown in the colored boxes. The data were from two TG monkeys and two WT controls, including 7–9 dendrites per monkey (unpaired Student's t test, *P < 0.05, n.s. no significance. Scale bar, 10 μm).

To determine whether the volume changes in the occipital lobe were due to altered cortical cytoarchitecture in the TG monkeys, we performed Nissl staining on the 2-year-old monkeys. A reduction in the number of cells was found in the layered structure of V1 cortex compared to the WT controls;however, no difference in cortical thickness was observed (Fig. 2A–C). After comparing the number of cells in layers 2–6 with those in the cortical plate (CP) layer, differences were found in the proportion of cells in layers 2–3 and layer 4 in the TG monkeys, with an increase in the proportion of neurons in layer 4 in the CP layer despite a lower overall number of neurons (Fig. 2D). It was proposed that layer 4, especially layer 4A, underwent additional modifications during recent human evolution [21]. These results suggest that TG monkeys likely mimic some of the human visual-related cortex phenotypes, which were not examined in previous studies of mice.

### Increased neuron numbers and delayed spinogenesis in TG monkeys

Our MRI findings pointed to a delayed pattern in brain development for the TG monkeys. To examine the potential relationship between *SRGAP2C* and embryonic brain development [14], we performed immunofluorescent staining at embryonic days 80 (E80), days 110 (E110) and days 133 (E133) by taking brain tissue samples near the central sulcus mostly belonging to the somatosensory region. Interestingly, compared to the WT controls, the TG monkeys only showed an increased number of CTIP2^+^ deep-layer (DL) neurons at E110, but not at E80 and E133. Neither did we see a difference in SATB2^+^ upper-layer (UL) neurons (Fig. 2E–I, Supplementary Fig. S6A and B). In light of previous mouse *in utero* electroporation (IUE) experiments, our results suggest that the TG monkeys exhibit accelerated cell migration, but this effect is limited to DL cells, not UL cells [14]. The CP neuron number increment is usually accompanied by cortex thickness expansion; however, we did not see this expected change in the cortical plate layer (Supplementary Fig. S6E and F). We also checked the progenitor marker Ki67 at E80, and observed a density increment in ventricular zone (VZ)/subventricular zone (SVZ) and inner subventricular zone (ISVZ), but not in the outer subventricular zone (OSVZ) (Supplementary Fig. S6C). Hence, the increase in cortical neurons at E110 is likely caused by the enhanced proliferation of progenitors in the germinal zone.

Spinogenesis is a vital process involving synapse formation, maintenance and activity-dependent elimination, essential for establishing neuronal networks and precise brain circuitry. Dendritic spines are vital for synaptic transmission and plasticity in excitatory neurons, and exhibit various shapes such as filopodia, thin, stubby and mushroom (Supplementary Fig. S6D) [22]. Expression of *SRGAP2C* in mouse cortical pyramidal neurons reportedly induces human-specific synaptic development traits, including increased synaptic density and prolonged maturation of excitatory and inhibitory synapses [14,23]. To examine the effect of *SRGAP2C* on dendrites and spines, using the fluorescent dye Alexa-Fluor-568 we visualized the neurons and their dendritic spines in the monkey brain slices sampled from DLPFC (Dorsolateral prefrontal cortex, area 9) and the primary somatosensory cortex I (S1) in concordance with the brain regions of the previous mouse studies (Fig. 2J) [15]. We analyzed two TG monkeys and two WT controls (Table 1) at 2 years old because this age represents the critical time for spinogenesis in monkeys as it is the end of the plateau phase of spinogenesis and the beginning of spine pruning [24,25].

The apical spine density of layer 2/3 (L2/3) PNs was significantly increased in the S1 region of the TG monkeys with only thin spines being increased. Although the apical spine density of L2/3 PNs in the prefrontal cortex (PFC) region did not show significant differences, a decrease in stubby spine was detected in the TG monkeys (Fig. 2K and L). Additionally, spinogenesis in the S1 region demonstrated a neotenic pattern similar to the reported *SRGAP2C* expression in the mouse S1, and there were less mature stubby spines in the PFC. In primates, the L2/3 PNs in the cortex are larger in size, with increased complexity (branching) and longer apical dendrites compared to those found in other mammalian species, contributing to an enhanced cortico-cortical connectivity [26]. The detected increase in cortico-cortical connections represents a distinctive human phenotype, which was also seen in the *SRGAP2C* humanized mice [15]. However, we did not see differences in arborization of dendrites in both L2/3 PNs of PFC and S1, as measured by Sholl analysis (Supplementary Fig. S6G–I). Collectively, these findings indicate that *SRGAP2C* leads to a delayed spinogenesis in the cerebral cortex, but with limited impact on dendrite arborization in TG monkeys.

### Embryonic transcriptome analysis of laminar brain tissue revealed molecular pathways underlying the DL neuron migration

**Figure 3.**
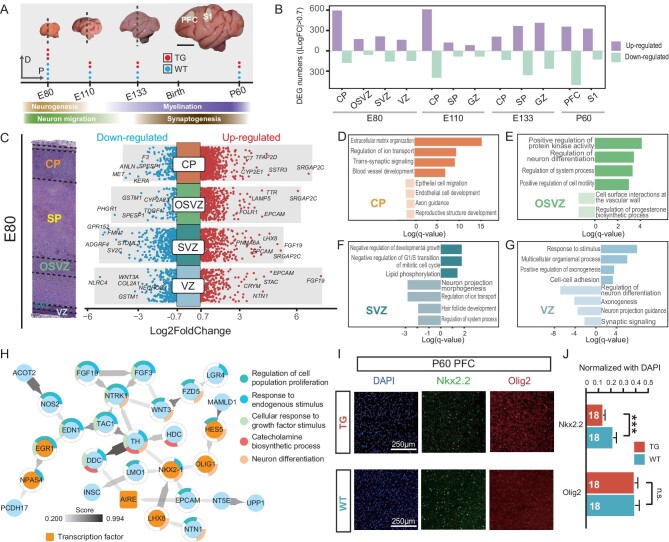
Transcriptome analysis of different brain laminae. (A) Schematic view of brain tissue samples at E80, E110, E133 and P60 (scale bar, 1 cm). (B) Counts of the up-regulated and down-regulated DEGs of different laminae in the TG monkeys. (C) Volcano plot at E80 showing DEGs of CP, OSVZ, SVZ and VZ. Left panel, the laminar structure of the monkey cortex at E80 (|log2FC| > 0.7, padj < 0.05). (D–G) GO enrichment terms of (D) CP, (E) OSVZ, (F) SVZ and (G) VZ at E80. (H) The PPI network of the up-regulated genes at E80 constructed using STRING. The outer circle depicts distinct colors indicating various GO enrichment terms. The color and thickness of the arrows reflect the interaction score. (I) Immunofluorescence staining for Nkx2.2 and Olig2, combined with DAPI, was performed using the WM samples of DLPFC at P60 (scale bar, 250 μm). (J) Quantification of Nkx2.2^+^ and Olig2^+^ cells normalized by DAPI^+^ at P60 in a 400 μm × 400 μm square region of the DLPFC WM. Two monkeys were analyzed for each group (TG or WT), covering nine regions per monkey (unpaired Student's t test, ***P < 0.001).

To investigate the molecular regulatory changes linked with fetal brain development in the TG monkeys, we conducted RNA sequencing (RNA-seq) of the brains at E80, E110 and E133. The left hemisphere brains were cryosectioned coronally near the central sulcus for laser microdissection (LMD) so that detailed profiling could be obtained from different laminae of the cortex. We dissected the cortex into four laminae at E80, including CP, OSVZ, SVZ and VZ. At E110 and E133, we dissected the cortex into three laminae, including CP, subplate (SP) and germinal zone (GZ, equivalent to the zone at E80 covering VZ, SVZ and OSVZ where the progenitor cells reside) (Fig. 3A and C).

We screened for differentially expressed genes (DEGs) between the TG monkeys and the WT controls in the corresponding laminae. In general, there were more up-regulated DEGs in the TG monkeys, especially in the CP lamina (Fig. 3B). The alteration of cellular composition plays a pivotal role in shaping human neural circuits [27]. For example, *SSTR3* is an up-regulated DEG in the CP at E80, and it is highly enriched in the cilia of excitatory neurons, with only occasional expression in inhibitory neurons. It is known that ciliogenesis in PNs is a key process in synaptic connectivity [28] (Fig. 3C). We also observed that *LAMP5*, a marker for the GABAergic neuron subsets originating from the non-medial ganglionic eminence (MGE) source, exhibited up-regulation in the OSVZ of the TG monkeys at E80 (Fig. 3C) [29].

At E80, distinct gene expression variations in excitatory and inhibitory neurons are evident within the CP and GZ layers. To construct the gene–gene interaction network at E80, we merged the RNAseq data of all laminae and screened for DEGs between the TG monkeys and the WT controls. A total of 126 up-regulated genes (log2FC > 0.7, padj < 0.05) were identified and then subjected to protein–protein interaction (PPI) network analysis using STRING software. The results showed that the largest PPI network contains 30 proteins (Fig. 3H). These proteins are predominantly associated with the regulation of cell proliferation and response to endogenous stimuli, such as TH, DDC and TAC1. In addition, there are proteins such as TH, DDC and HDC involved in the catecholamine biosynthetic process, suggesting that *SRGAP2C* may influence cellular composition by modulating the composition of interneurons.

Given the detected increase in DL neurons at E110, we investigated the mechanisms involved—whether it is due to enhanced migration, increased cell differentiation or a combination of both. We firstly analyzed differential gene expression in the VZ and SVZ at E80, as this stage might contribute to DL neuron proliferation. We found elevated expression of *FGF19* in the TG monkeys. *FGF19* plays a crucial role in zebrafish forebrain development and the FGF19 protein promotes cell cycle exit and neuronal differentiation in cultured human neural progenitor cells [30,31]. Furthermore, we found the SVZ up-regulated DEGs enriched in negative regulation of development growth (log(q-value) = −1.790) and negative regulation of the G1/S transition of the mitotic cell cycle (log(q-value) = −1.790) (Fig. 3F). The timing of mitotic cycle exit is a major mechanism related to progenitor pool expansion, which may explain the increase in the CTIP2^+^ neurons at E110 in the TG monkeys (Fig. 2H).

During neural cell migration starting from the VZ, extracellular matrix remodeling and ciliary movement are crucial factors [32]. In our differential gene expression analysis in multiple brain laminae, including the OSVZ, SP and CP, we found that at E110, all laminae (GZ, SP and CP) exhibited gene ontology (GO) enrichment in the extracellular matrix organization in TG monkeys (Supplementary Fig. S7B–D), whereas this was not observed at E133 (Supplementary Fig. S7F–H). This suggests that E110 represents a critical period for migration, and the elevated expression of extracellular matrix (ECM)-related genes potentially contributes to the acceleration of DL neuron migration. For example, *EPCAM* is highly expressed in the GZ (OSVZ, SVZ, and VZ) of TG monkeys, and encodes a transmembrane protein in the extracellular matrix that influences actin organization associated with cytoskeletal organization, cell proliferation and cell migration [33]. It is involved in regulating RhoA signaling, possibly as a consequence of the introduced *SRGAP2C* in the TG monkeys. Collectively, these results indicate that the coordinated regulation of both processes (neurogenesis and migration) leads to an augmentation in the DL neurons at E110.

At E133 (Supplementary Fig. S7A and E) in the CP and SP laminae of the TG monkeys, the up-regulated genes showed enrichment in oxidative phosphorylation pathways (Supplementary Fig. S7B–D and F–H). To visually represent these enriched pathways (q-value < 0.05), word cloud representations were generated for both up-regulated and down-regulated genes at the CP of E110 and E133 (Supplementary Fig. S7I and J). These representations indicated a time shift of up-regulated genes and down-regulated genes.

Collectively, these data suggest that *SRGAP2C* regulates embryonic cell migration and differentiation, influences cellular composition, and ultimately modifies brain circuitry connections by orchestrating the laminar-related gene networks in TG monkeys.

### Postnatal transcriptome analysis suggests delayed myelination in the PFC of TG monkeys

The postnatal brain development of primates is a crucial and intricate process that sculpts the nervous system. The transcriptomic signatures after birth are associated with neurodevelopmental processes, such as synaptogenesis, astrogliogenesis and myelination [34]. We screened DEGs of the PFC and S1 brain regions at P60, a pivotal time point for establishing neural circuits. We detected 122 up-regulated genes and 505 down-regulated genes in the PFC, and 328 up-regulated genes and 126 down-regulated genes in S1 (Supplementary Fig. S7K and L).

The down-regulated DEGs in the PFC are mostly enriched in glial cell differentiation, oligodendrocyte differentiation and ensheathment of neurons. We reasoned that these might be consistent with the MRI data in which we saw a volume reduction of the prefrontal cortex WM (Supplementary Fig. S2C). It is known that myelination plays a crucial role in establishing brain connectivity, enabling rapid information transfer and supporting higher-order cognitive functions, and human neocortical myelination is developmentally protracted compared to chimpanzees [35]. We utilized two marker genes to visualize the cells involved in myelination, including *Nkx2.2* (mostly labels OPC under differentiation) and *Olig2* (widely labels oligodendrocyte). The immunofluorescent results showed that in the DLPFC WM of the TG monkeys, there were fewer *Nkx2.2^+^* cells compared with the WT controls, while no change was detected for the *Olig2^+^* cells (Fig. 3I and J). This pattern is consistent with prior studies, as oligodendrocytes differentiate and produce myelin proteins with rapid down-regulation of *Nkx2.2* expression, leading to inhibition of myelin basic protein (MBP) gene expression *in vitro* [36,37]. Overall, these results show that in the neocortex, *SRGAP2C* causes down-regulation of the ensheathment genes, resulting in delayed (the so-called neotenic) myelination in the PFC.

### Longitudinal transcriptome analysis confirms the neotenic pattern in TG monkeys

**Figure 4.**
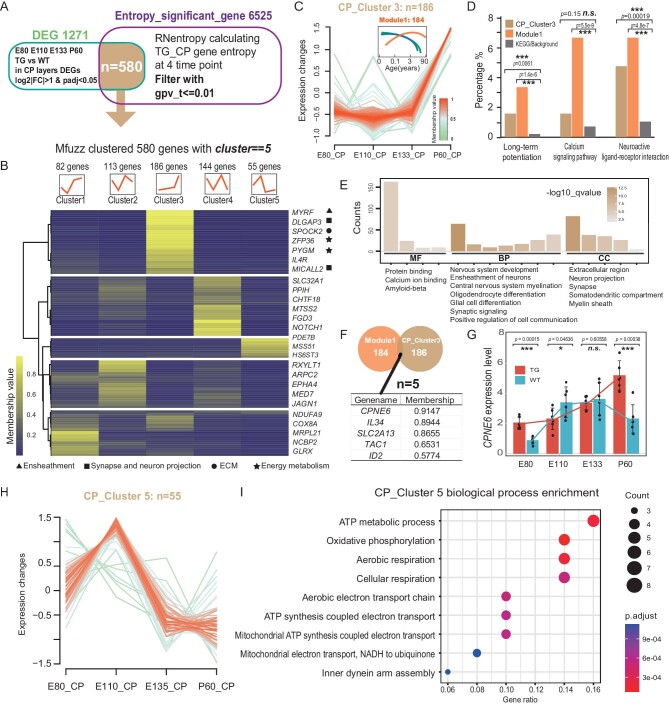
Longitudinal transcriptome analysis reveals a neotenic gene expression pattern in the TG monkeys. (A) The schematic view of the procedure of filtering the temporal DEGs (tDEGs) in the CP. (B) Heatmap showing the expression patterns of the classified five clusters. (C) The temporal expression pattern of genes in CP Cluster-3. The gradient color indicates the range of gene membership values. The embedded graph is the temporal expression pattern of Module 1. The orange line shows the neotenic human expression tendency, modified from *Liu et al.* [40]. (D) Comparison of the percentages of genes in CP Cluster-3, Module 1, and the background control that is associated with the three KEGG pathways. The *P*-value indicates whether the percentage of KEGG-pathway-related genes is significantly enriched in the gene sets compared with the background control (hypergeometric test, ***P < 0.001, n.s. no significance). (E) GO enrichment gene counts of CP Cluster-3, covering molecular function (MF), biological process (BP) and cellular component (CC). (F) The five overlapped genes between Module 1 and CP Cluster-3. (G) Validation of the *CPNE6* expression level by RT-qPCR (two-tailed Students’ t test, *P < 0.05, ***P < 0.001, n.s. no significance). (H) The temporal expression pattern of genes in CP Cluster-5. The gradient color indicates the range of gene membership values. (I) GO enrichment gene counts of the biological process (BP) of CP Cluster-5.

Temporal variations in brain development may conceivably exhibit an association with innovative cognitive maturation patterns in primates [34]. We integrated the 19 transcriptomes at 4 prenatal and postnatal time points. We calculated the gene entropy by RNentropy to describe the genes with significant changes in the CP DEGs over time [38], and the 580 identified CP DEGs were classified into 5 clusters by Mfuzz calculated membership value [39]. These DEGs were defined as the CP temporal DEGs (tDEGs) with distinctive expression trajectories (Fig. 4A). In particular, in Cluster 3, there are 186 genes showing a ‘neotenic’ expression pattern (Fig. 4B and C). A previous study has validated several expression modules corresponding to human-specific brain phenotypes such as enhanced synaptic transmission, ion transport and cell–cell communication [40]. For example, Module 1, classified as expression-delayed genes, showed a human-specific shift in the timing of cortical spinogenesis (Fig. 4C). The kyoto encyclopedia of genes and genomes (KEGG) enrichment of Cluster 3 showed a similar pattern to Module 1, confirming the neotenic expression pattern of the involved pathways (Fig. 4D). In other words, these genes have a delayed up-regulation in the CP of TG monkeys. More importantly, the CP tDEGs with the highest membership values are mostly related to ensheathment, synapse and neuron projection, ECM, and energy metabolism, and the GO analysis showed similar categories of enrichment (Fig. 4B and E).

Next, we looked at the overlapped genes between Cluster 3 and the reported Module 1, and we identified five such genes, including *CPNE6, IL34, SLC2A13, TAC1*, and *ID2. CPNE6*, with the highest membership value, was highly expressed in the TG monkeys after birth (Fig. 4F). Previous data showed that it is highly expressed in postnatal excitatory neurons and its expression is associated with the size of postsynaptic spine heads [41,42]. We performed quantitative reverse transcription PCR (RT-qPCR) (from E80 to P60) and validated the delayed expression peak of *CPNE6* in the TG monkeys, and similar patterns were detected for the other two genes (*SLC2A13* and *ID2*) (Fig. 4G, Supplementary Fig. S8A and B).

As mentioned previously, E110 appears to be the pivotal time point associated with an increase in the number of DL neurons, potentially accompanied by a corresponding expression pattern at the transcriptome level. Interestingly, Cluster 5, distinguished by its notably high expression, particularly during the prenatal stage at E110, caught our attention (Fig. 4H). Cluster 5 contains a relatively small number of genes (55); however, it significantly enriches genes associated with mitochondrial oxidative phosphorylation (gene counts: 8/55) (Fig. 4H and I). Metabolism and mitochondria play pivotal roles in governing cell fate transition and maturation within the brain [43]. It has been reported that the relatively slow metabolic rate in human mitochondria extend the maturation window of neurons [44]. Hence, these genes in Cluster 5 exhibit a pattern of accelerated expression in the TG monkeys, suggesting that there is substantial oxidative phosphorylation accompanying cell proliferation and differentiation. The precipitous decline in the expression of genes within Cluster 5 from E133 onwards, extending into the postnatal period, could conceivably be associated with the observed delay in synaptogenesis and myelination within the TG monkeys.

Furthermore, we performed the same analysis on the GZ genes and validated 662 GZ tDEGs throughout three time points, E80, E110, and E133 (Supplementary Fig. S8C and D) since GZ does not exist at P60. The cluster 2 of the GZ tDEGs, including 408 genes, also showed a neotenic (the relative expression level is <0 at E80 and E133) tendency and the most significant signs of GO enrichment include calmodulin binding, synaptic signaling and synapses (Supplementary Fig. S8E and F). There are 15 genes overlapping between GZ Cluster 3 and Module 1. The *SCN2A* gene, with the highest membership value, encodes NaV1.2, a neuronal sodium channel protein primarily found in excitatory neurons throughout the brain. E133 is typically considered the onset of oligodendrogenesis, and the expression of *SCN2A* influences oligodendrocyte development and excitability in the mammalian brain, while its relatively low expression at the E133 epoch might lead to subsequent postnatal myelination delays owing to its protracted expression [45].

In summary, our gene expression analysis across embryonic and postnatal stages revealed unique patterns in the TG monkeys within the CP and GZ. These patterns, including accelerated and delayed expression, were linked to the increase in DL neurons at E110. In addition, we identified candidate genes that are shared with the reported human neotenic module and the expression patterns of these genes may explain the delayed synaptic development and myelination in postnatal TG monkeys.

### General behavior analysis and test of hand flexibility

To explore the phenotypic effects resulting from the observed molecular and histological changes in the TG monkeys, we first conducted a general behavioral assessment, which included video recordings of their daytime activities and night-time sleep (four TG versus four WT age-matched monkeys, 24–36 months old; see the ‘Methods’ section for details). To analyze daytime activities, we categorized the behavior of monkeys into three groups: self-injuring behaviors, stereotypical behaviors (including licking/biting bars, rubbing bars, twirling/rocking, hand stereotype, saluting/eye poking, self-clasping and floating limb) and daily-normal behaviors (including locomotion, resting and self-grooming) [46,47]. We recorded the number and length of each behavior that happened within an hour. To analyze night-time sleep, we studied the amount of time spent awake and asleep during the night [48]. The results revealed that there was no significant variation in self-injuring behaviors and stereotypical behaviors between the two groups (Supplementary Table S8), implying that *SRGAP2C* had no effect on the mental state of the TG monkeys. Interestingly, the resting and locomotion frequencies of the TG monkeys were significantly lower than those of the WT controls, though the duration of both states was not significantly different (Supplementary Table S8). Similarly, although there was no significant difference between the two monkey groups in terms of waking duration and sleep duration during nocturnal sleep, the TG monkeys had a significantly lower number of awakenings during sleep (Supplementary Table S8). These phenomena indicate that the TG monkeys tend to have fewer transitions between different states.

**Figure 5.**
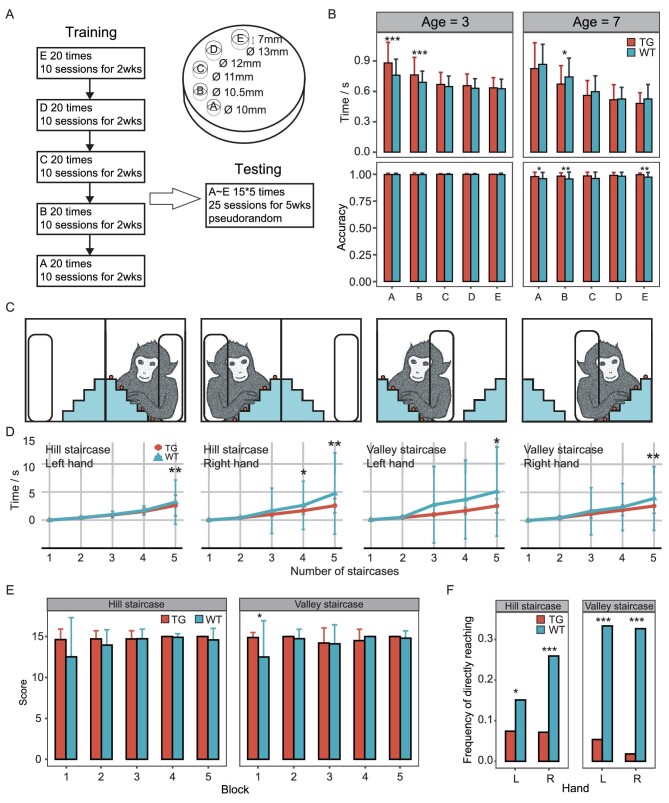
Tests of general behavior and motor planning and execution skills. (A) Diagram of the Kluver board task. (B) The time taken (top) and the success rate (bottom) (mean ± standard error) of the successfully retrieved food rewards from each hole on the board. Student's t test, *P < 0.05, **P < 0.01, ***P < 0.001. (C) Diagram of the Hill-and-Valley Staircase task. (D) The cumulative time taken to successfully retrieve food rewards with monkeys’ left or right arms. Student's t test, *P < 0.05, **P < 0.01. (E) The total scores achieved by the monkeys in each experiment. The data from the left hand and the right hand were added up for each monkey. The peanut closest to the slot was assigned one point and the farthest was assigned five points. Mann-Whitney U-test, *P < 0.05. (F) Comparison of the frequencies of reaching directly for food rewards behind the organic glass plate, where the TG monkeys displayed significantly lower frequencies for both the hill and valley staircase. Chi-square test, *P < 0.05, ***P < 0.001.

To evaluate the monkeys’ finger flexibility and complex motor planning and execution skills, we conducted the Kluver board task [49,50]. The Kluver board task allows monkeys to extract food from five neighboring holes with different diameters. The time taken and the success rate were recorded (Fig. 5A). Tests were done on monkeys at two developmental stages, 3–4 years old and 7–8 years old. The results indicated that the TG monkeys had a longer feeding time than the WT controls when they were 3–4 years old; however, when they reached 7–8 years old, the TG monkeys completed the test faster with a greater success rate (Fig. 5B, Supplementary Fig. S9C).

Next, we applied the Hill and Valley Staircase task. Monkeys must put their right arm through the holes in the organic glass board and gather the peanut kernels from the five stairs behind it, which are configured in the form of ‘Λ’ or ‘V’ (Fig. 5C). When food rewards are successfully obtained, points are gained. During the monkey testing phase, we monitored the scores and the cumulative time taken to remove each food particle. The results indicated that the TG monkeys had higher scores and shorter feeding times than the WT controls (Fig. 5D and E, Supplementary Fig. S9D), suggesting enhanced complex motor planning and execution skills. Having monkeys retrieve food pellets from behind a transparent barrier was also used to examine cognitive performance, as it requires the monkeys to inhibit the predominant motor response of directly reaching for a food item that is placed behind a transparent barrier and instead apply a successful response to bypass the barrier [51]. Hence, this result suggests that the TG monkeys likely acquired enhanced cognitive skills since they did not directly reach for food as often as the WT controls did (Fig. 5F).

## DISCUSSION

The introduction of the *SRGAP2C* transgene in the brain of the TG monkey mimics several important features related to human brain connectivity, such as increased cell abundance in layer 4 of the visual cortex, the delayed maturation of synapses and myelination, the shifted (neotenic) transcriptomic pattern, and the left-hemisphere lateralization of the cortical region associated with hand flexibility improvement. These detected changes suggest the critical role of *SRGAP2C* in shaping human brain development and function.

### Increased DL neurons and synapse density in TG monkeys

Previous studies have provided limited evidence regarding the involvement of *SRGAP2C* in neurogenesis. We observed augmentation in the number of DL neurons during corticogenesis mediated by *SRGAP2C*, whereas no significant change was detected in the number of UL neurons. Consistent with these findings, a prior investigation demonstrated that the electroporation of *SRGAP2C* in mouse cortex progenitors increased the rate of radial migration, as assessed by the proportion of neurons successfully reaching the CP within 4 days [14]. Compared with mice, the presence of complicated CPs and the extended corticogenesis window in TG monkeys allows for a more comprehensive understanding of the precise pattern of neuron migration. Accordingly, we identified an increase in DL neurons at E110 and an elevation in VZ/SVZ mitotic cell density at E80 in the TG monkeys, while no significant differences were observed in OSVZ. The heightened abundance of mitotic cells in VZ/SVZ at E80 in the TG monkeys is likely responsible for the detected increase in the generation of DL (layer 5) neurons, which may eventually enhance the connections between the cortical region and the subcortical areas. However, the increase in DL neurons induced by *SRGAP2C* did not contribute to the thickening of the CP, which is substantially amplified in humans compared to non-human primates and rodents [14,52].

As *SRGAP2C* is one of the hub genes related to the human-specific features of PNs, we studied the morphology of layer 2/3 neurons, which play a pivotal role in cortico-cortical circuitry [53]. Our findings revealed a significant increase in the total number of spines of layer 2/3 PNs, especially in the S1 of TG monkeys, while no increase was seen in the PFC. Furthermore, the observed increase in thin spines within the S1 region and the decreased stubby spines in the PFC collectively imply an extended maturation process of dendritic spines. This contrasts with the findings from the *SRGAP2C* mouse model, where only the S1 region exhibited heightened apical dendritic spine density [14]. Contrary to our initial expectations, we did not observe significant changes in dendritic arborization in the layer 2/3 PNs of TG monkeys. This is different from the pattern seen in the mouse model, which displayed enhanced cortico-cortical connectivity related to layer 2/3 neurons [14]. Such discrepancies might arise from differences in animal models and/or measurement techniques. Our study unveils a delayed spinogenesis in layer 2/3 PNs in both the PFC and S1. Analysis of the morphology of these neurons suggests that the increased connectivity in the TG monkeys primarily results from alterations in dendritic spine density rather than dendritic structure modifications.

### Delayed transcriptome expression in TG monkeys suggests human-like neoteny during brain development

The shifted patterns of genes expressed spatiotemporally offer the potential to reshape the brain connectivity of TG monkeys. To identify the transcriptomic changes brought about by *SRGAP2C* during corticogenesis, spinogenesis and myelination, we conducted a comprehensive temporal transcriptome analysis. We integrated data from E80 to P60, and we revealed the role of *FGF19* in radial neuronal migration toward the appropriate position in the CP. Additionally, we observed the involvement of *EPCAM* in the VZ/SVZ at E80 and the expression of ECM-related genes in the CP and SP laminae at E110 and E133. These orchestrated processes led to a sequential increase in DL neurons at E110. Given that previous studies have emphasized the significance of the ECM proteins in neural progenitor self-renewal [54], our findings highlight the primary role of the ECM in providing essential niches for neuron migration. Furthermore, we found that TH (tyrosine hydroxylase), a rate-limiting enzyme in catecholamine (including dopamine) biosynthesis, was up-regulated as a network hub-gene in the TG monkeys. It was reported that the specialized expression of TH in humans can lead to an increase in dopamine levels and improve the plasticity of dopaminergic interneurons, and eventually contribute to the increased local connections between excitatory neurons [55]. Additionally, our temporal transcriptome analyses consistently demonstrated a prolonged pattern of neotenic gene expression in the TG monkeys. In particular, we identified *CPNE6*, a gene associated with synaptogenesis, and its expression is likely modified by the transgene *SRGAP2C*. Overall, the differential expression of these networks may contribute to the elaboration of signaling pathways within neurons, neuronal and synaptic ultrastructural components, and even cellular composition.

### Increased regional connectivity in TG monkeys

In addition to the localized alterations in circuitry attributed to variances in cellular composition and synaptic connectivity density, changes in brain connectivity ultimately manifest across interregional connections and may be concomitant with potential enhancements in higher cognitive function. The preceding study has documented an association between behavioral flexibility and alterations in both the structure and function of the frontal cortical network of macaques [56]. The delayed myelination of the human brain plays a pivotal role in extending the window for shaping neural network plasticity, which is notably distinct from the accelerated neocortical myelination development observed in chimpanzees [27]. Our data showed a delayed myelination of DLPFC in the TG monkeys, and subsequent postnatal transcriptome analysis revealed down-regulation of genes enriched in oligodendrocyte and ensheathment pathways. Consistently, we detected fewer OPCs in the DLPFC WM of TG monkeys.

Hemispheric lateralization is a key aspect of human brain organization, with notable disparities in both structure and function [57]. For instance, the left planum temporale, known as Wernicke's area, is active in tasks related to auditory processing and receptive language [58]. We demonstrated that the temporal-lobe-related cortex WM and WM tracts exhibited a left-hemispheric difference between the TG monkeys and the WT controls. The pathway constituted by superior temporal gyrus, inferior temporal gyrus WM, and uncinate fasciculus tract is considered as a crucial circuit for social behavior in monkeys, and is believed to share evolutionary origins with human communication [20]. Remarkably, we detected a reduction in occipital lobe volume in the TG monkeys compared to the WT controls. Furthermore, differences in myelination were also observed in the WM tracts connecting to the visual pathways, such as the inferior longitudinal fasciculus and optic tract. Our subsequent experiment confirmed an increase in the abundance of layer 4 neurons in the visual cortex of the TG monkeys, mirroring the organization of the visual cortex cells in humans [59]. We therefore speculate that *SRGAP2C* may influence macroscopic cortical connectivity, which is specialized in human brain evolution.

We employed relatively simple tests when accessing the general behavior and complex motor planning and execution skills of the TG monkeys, since complicated tasks such as the delayed-matching-to-sample task for testing working memory proved to be too difficult for cynomolgus monkeys. Interestingly, in the general behavior test, the TG monkeys displayed reduced between-state transitions in resting/locomotion and sleep/awake. How these behavioral changes are connected with the observed brain connectivity changes is yet to be explored. In addition, in the test of finger flexibility, we saw enhanced motor planning and execution skills in the TG monkeys, consistent with the expected benefits of the neotenic brain development that is believed to provide an extended window of neural plasticity. Nevertheless, more cognitive tests are needed to see how *SRGAP2C* contributes to human-like cognitive enhancement in TG monkeys. Finally, it should be noted that we used synapsin-promoter (a neuron-specific promoter) in the TG monkey model. Theoretically, we cannot rule out the possibility that if expression of *SRGAP2C* were driven from its own promoter, the phenotype of the TG monkeys might have shown some differences.

In summary, we generated a TG monkey model of *SRGAP2C*, the human-specific gene copy. The TG monkeys carrying *SRGAP2C* showed modified cortical connectivity and developmental changes, including increased DL neuron density, delayed postnatal myelination, delayed layer 2/3 neuron spinogenesis and improved brain region connectivity. Cognitively, we observed improved complex motor planning and execution skills in the TG monkeys.

## MATERIALS AND METHODS

Detailed materials and methods are available in the Supplementary Data.

## Supplementary Material

nwad281_Supplemental_Files
