# Supplementary material for: Brain developmental and cortical connectivity changes in transgenic monkeys carrying the human-specific duplicated gene SRGAP2C
Source: Natl Sci Rev. 2023 Nov 3;10(11):nwad281. doi: 10.1093/nsr/nwad281 (PMC10712708; doi:10.1093/nsr/nwad281)
Supplement: nwad281_Supplemental_Files [file nwad281_supplemental_files.zip › Supplementary_figures.pdf]

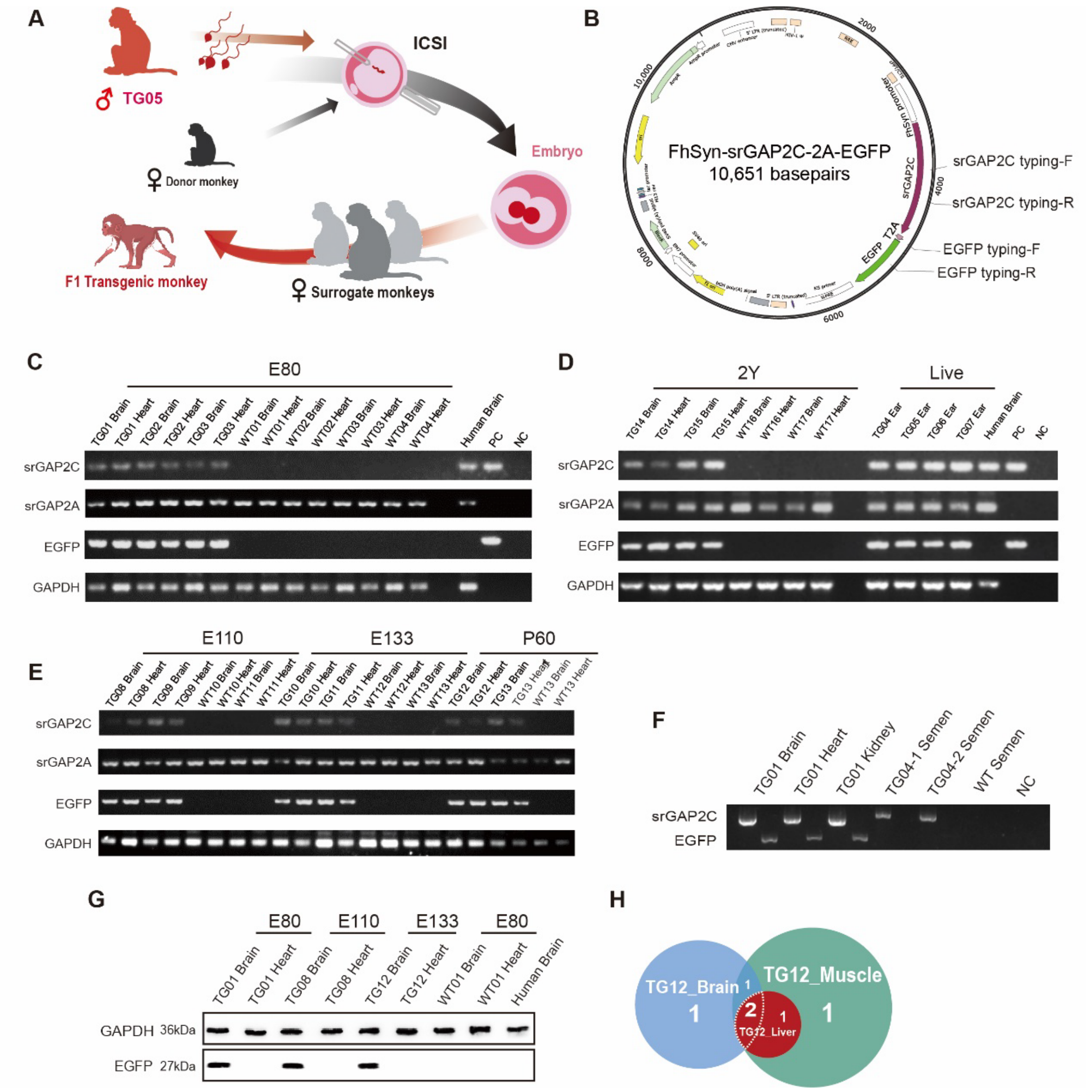

Figure S1. Generating TG monkeys and regional-specific maturation patterns revealed by longitudinal MRI. (A) Schematic illustration of the generation of the transgenic monkeys. **(B)** Plasmids map of FhSyn-srGAP2C-2A-EGFP as shown in Fig.1B with typing primer pairs used in this study. **(C-E)** The presence of the transgene *srGAP2C* and *EGFP* in brain and body tissue of the transgenic monkeys (TG01-TG15) was confirmed by PCR**. (C)** TG01-TG03; **(D)** TG04-TG07, TG14 and TG15; **(E)** TG08-TG13; PC, positive control, plasmid FhSyn-srGAP2C-2A-EGFP; NC, negative control, ddH2O. **(F)** Presence of the transgene *srGAP2C* in transgenic monkeys (TG01) and semen of TG05 used for ICSI to obtain F1 transgenic monkeys; NC, negative control, ddH2O. **(G)** Western blot analysis confirmed the expression of EGFP proteins in brain and heart tissues. **(H)** The *srGAP2C* copy numbers overlapped between the brain and body tissue of TG12 showed more CNs in the brain.

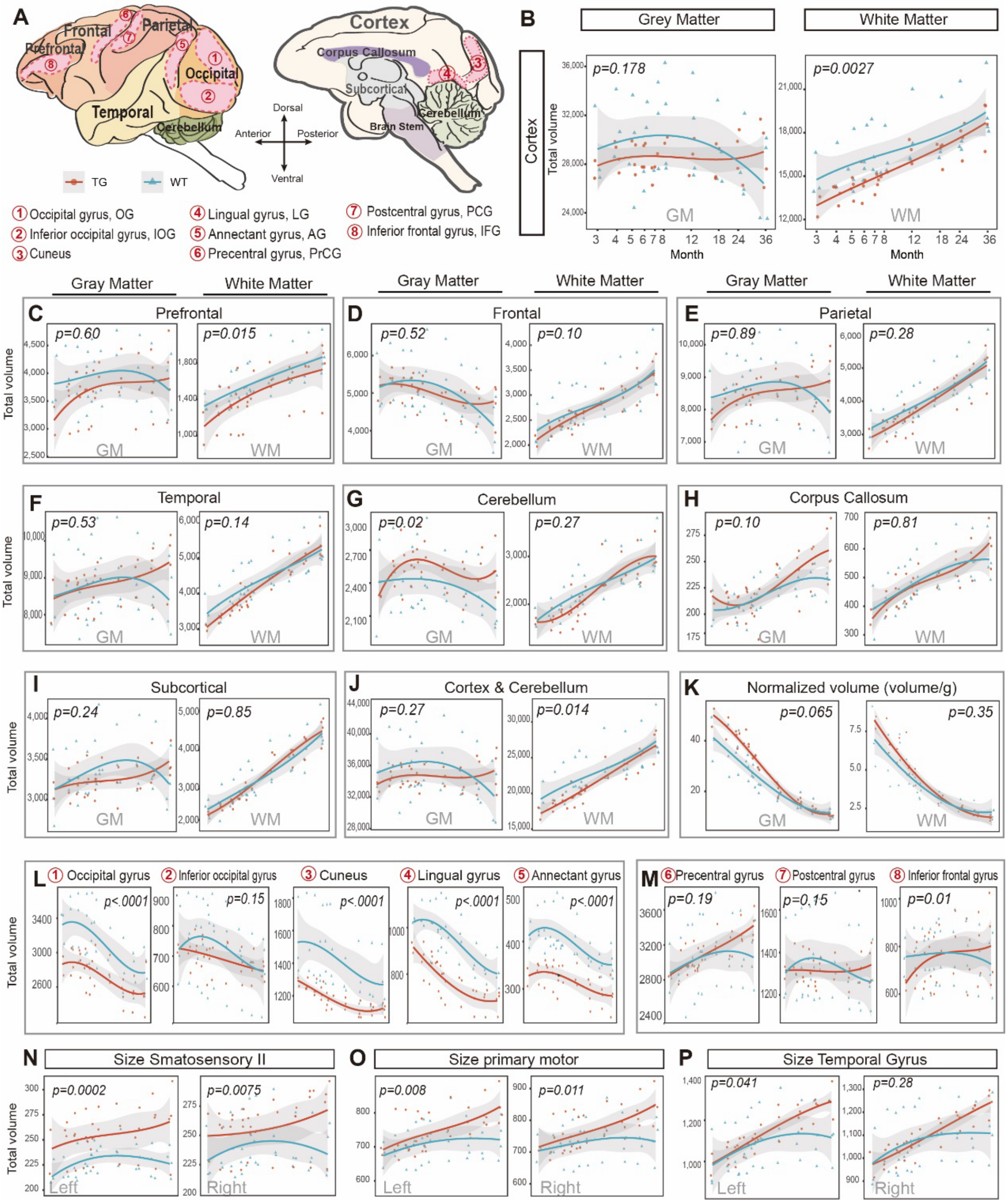

Figure S2. **The gray matter and white matter volumes after partitioning.** **A**. The schematic of brain parcellations, numbers within the circle represent more specific divisions from Huangatlas; **B-K**. Total volume based on MRI T1 data, left, gray matter volume, right, white matter volume **(B)** Total cortex size, **(C)** Prefrontal lobe, **(D)** Parietal lobe, **(E)** Frontal lobe, **(F)** Temporal lobe, **(G)** Cerebellum, **(H)** Corpus Callosum, **(I)** Subcortical region, **(J)** cortex and cerebellum **(K)** Normalized volume by weight (g). **L-M**. The total volume of detailed parcellation of (**L**) occipital region and (**M**) parietal region; **N-P**. Total volume of detailed parcellation of **(N)** somatosensory II **(O)** primary motor cortex **(P)** temporal gyrus, left, left-hemisphere, right, right-hemisphere; Group effect p-value was calculated based on LMM (linear mixed model), and p<0.05 was taken as statistically significant.

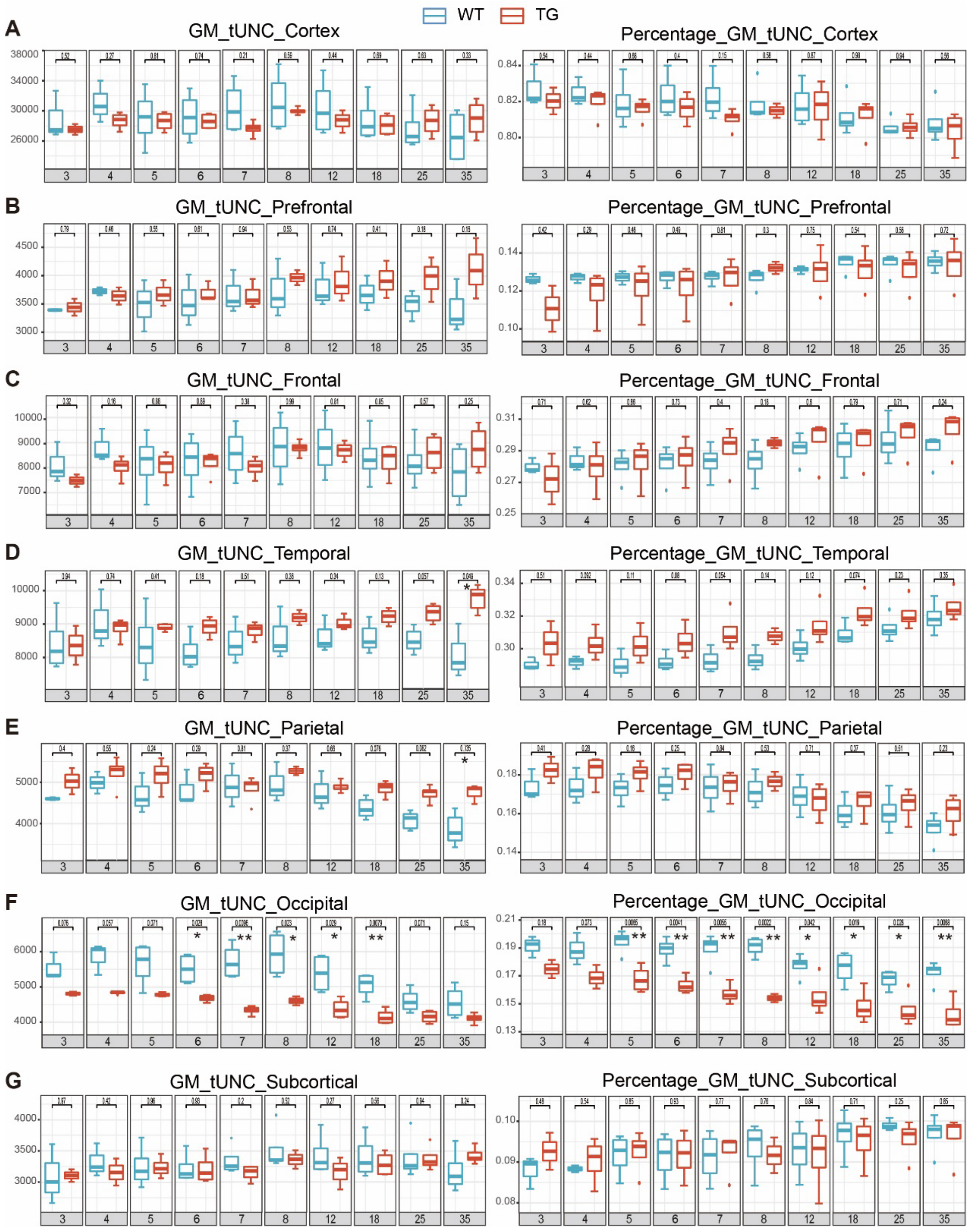

Figure S3. Gray matter volume trend and percentage relative to total volume. (A) Total cortex size; **(B)** Prefrontal lobe; **(C)** Frontal lobe; **(D)** Temporal lobe; **(E)** Parietal lobe; **(F)** Occipital lobe; **(G)** Subcortical region; p-value is calculated by un-paired Students' t-test at each time point between groups. * p<0.05, ** p<0.01.

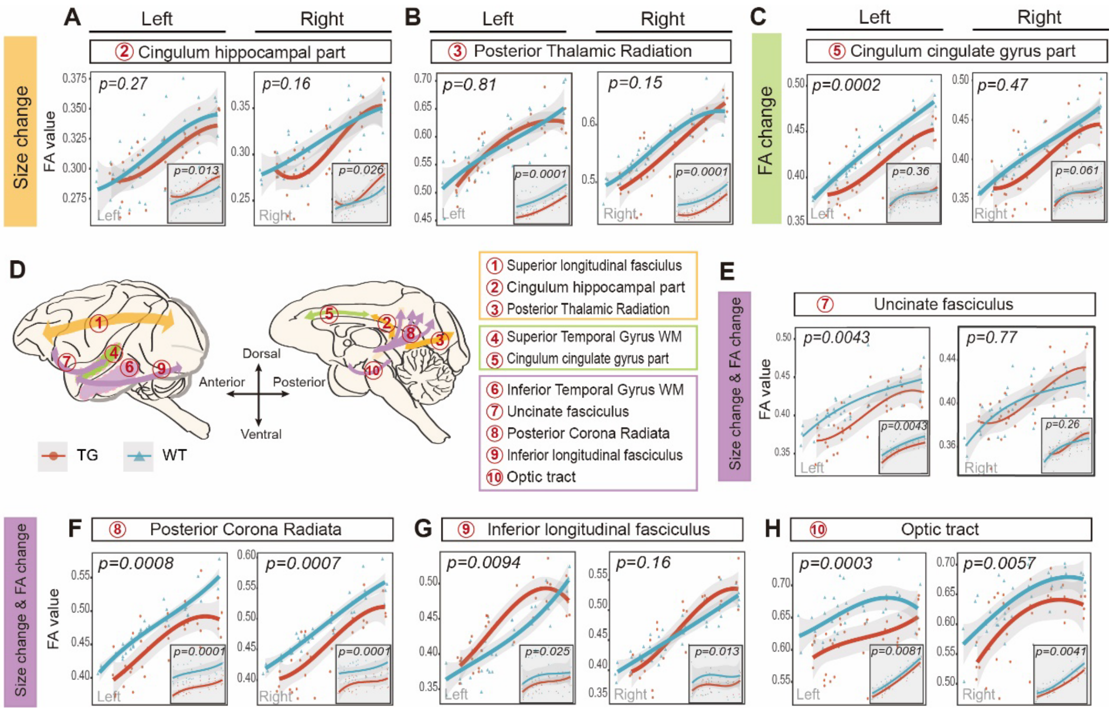

Figure S4. Differences in T1 size and DTI FA-value in various white matter tracts. A-B. Parcellations of WM with T1-size differences (embedded figures) but no FA value difference. **(A)** Cingulum hippocampal part; **(B)** Posterior thalamic radiation; **C.** Parcellations of WM with T1-size no differences (embedded figures) but FA value difference. Cingulum cingulate gyrus part ; **D.** The schematic of brain white matter precision parcellations; **E-H**. Parcellations of WM with both T1-size (embedded figures) and FA value show differences. **(E)** Uncinate fasciculus; **(F)** Posterior corona radiate; **(G)** Inferior longitudinal fasciculus; **(H)** optic tract; Group effect p-value was calculated based on LMM (linear mixed model), and $p<0.05$ was taken as statistically significant. left, left hemisphere, right, right hemisphere;

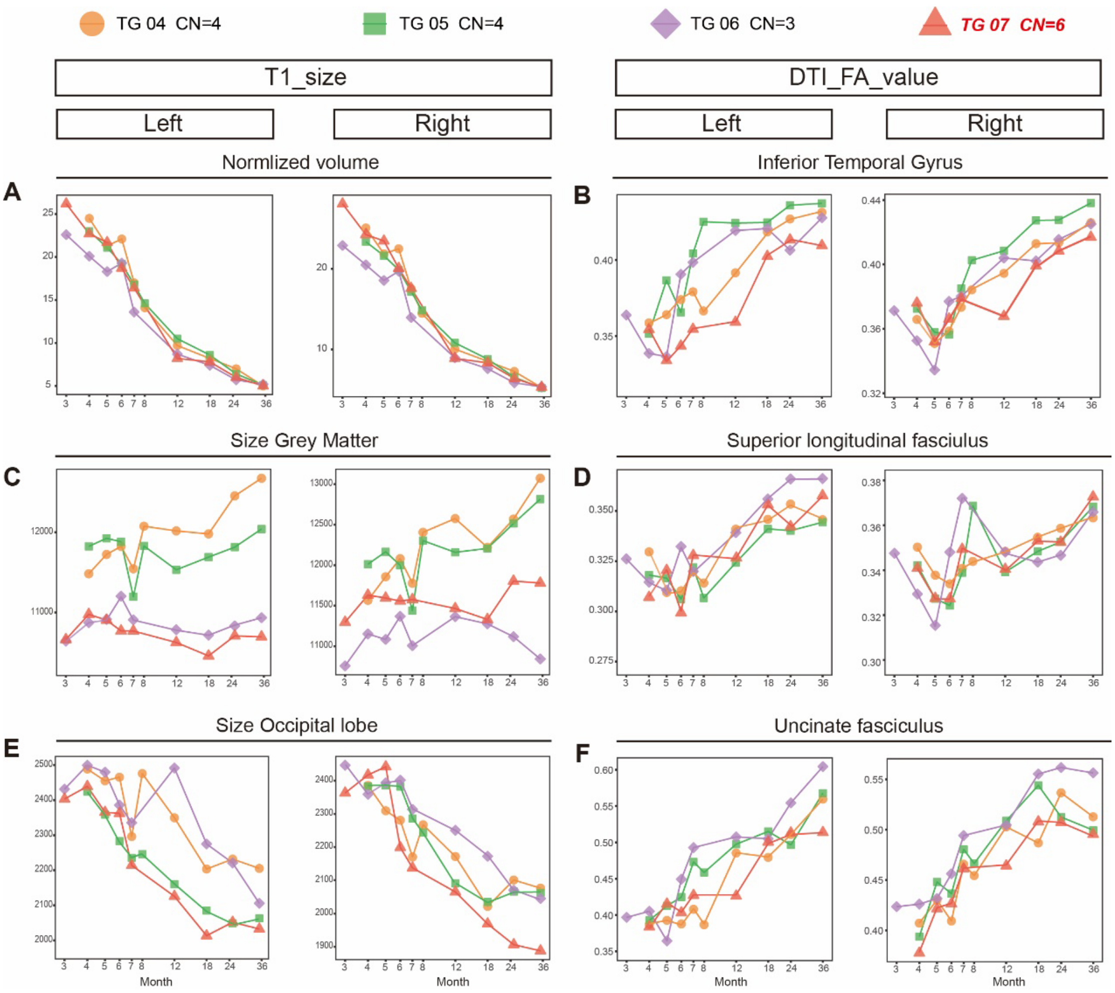

Figure S5. Correlation between the *srGAP2C* copy numbers and MRI data. (A) Normalized total brain volume; **(B)** FA-value of inferior temporal gyrus; **(C)** T1-based total gray matter size; **(D)** FA-value of Superior longitudinal fasciculus; **(E)** T1-based size of occipital lobe; **(F)** FA-value of uncinate fasciculus. CN, copy numbers; left, left hemisphere; right, right hemisphere; TG07, TG monkey with the highest copy numbers.

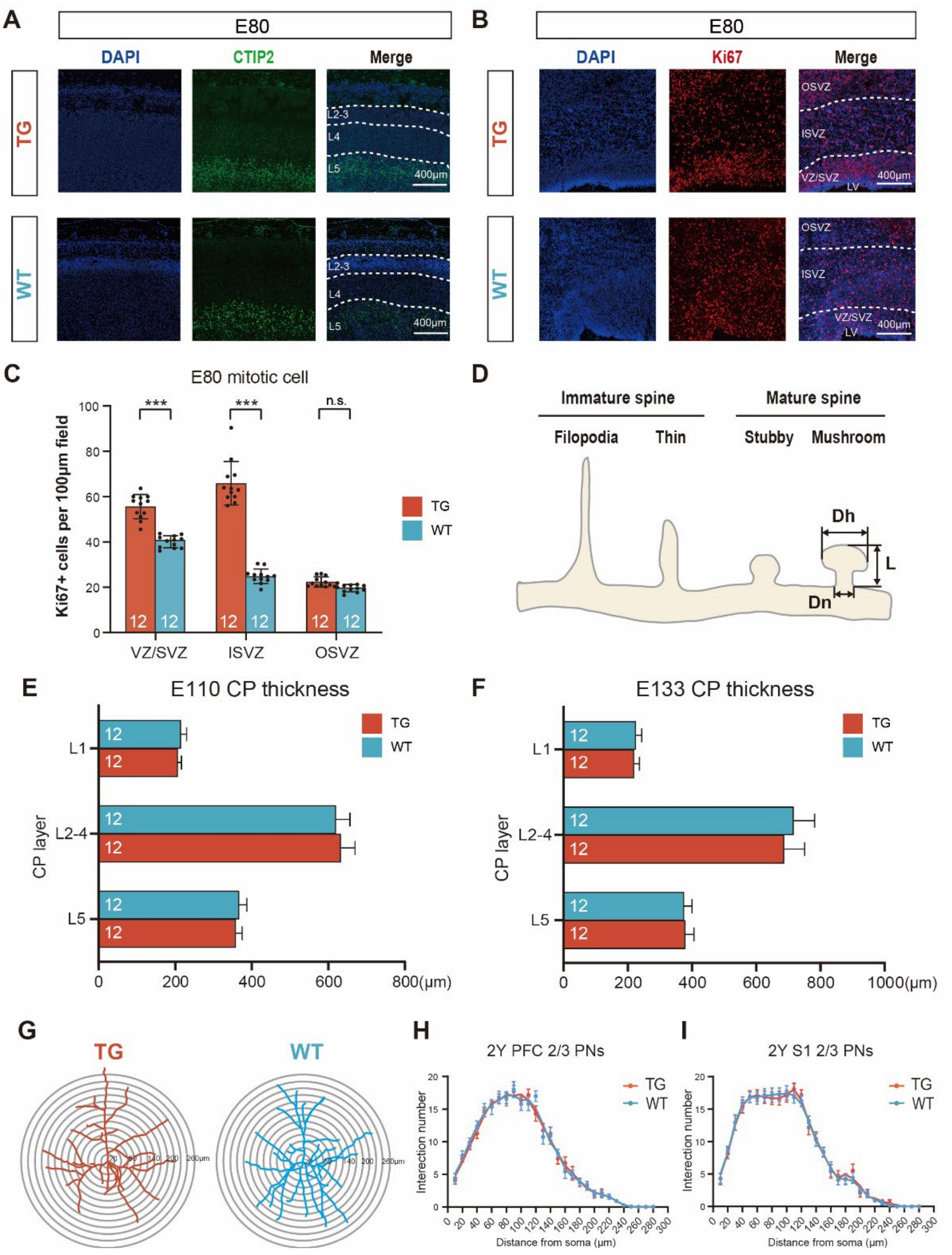

Figure S6. Immunofluorescence of embryonic samples and sholl analysis of 2/3 PNs. (A) Immunofluorescence for CTIP2 (green), combined with DAPI (cyan) of E80 cortical plate. (Scale bar, 400μm); **(B)** Immunofluorescence for Ki67 (red) represents the mitotic cell, combined with DAPI (cyan) of E80, LV, and lateral ventricle. (Scale bar, 400μm); **(C)** Quantification of Ki67+ neurons at E80 in a 100-μm wide region of TG and WT germinal zone, 12 sections per group (unpaired Student's t-test, *** p<0.001); **(D)** Schematic of spine subtypes and spine size measurements; **(E-F)** Measurements of cortical plate layer thickness, **(E)** E110 and **(F)** E133, L1, layer1 L2-4, layer2-layer4, L5, layer5 (unpaired Student's t-test); **(G)** Representative morphology of layer 2/3 pyramidal neurons of S1; **(H-I)** Sholl analysis quantification of layer 2/3 pyramidal neurons at 2-year-old monkeys. **(H)** PFC **(I)** S1 PN, pyramidal neuron. (unpaired Student's t-test, bar, 95% CI).

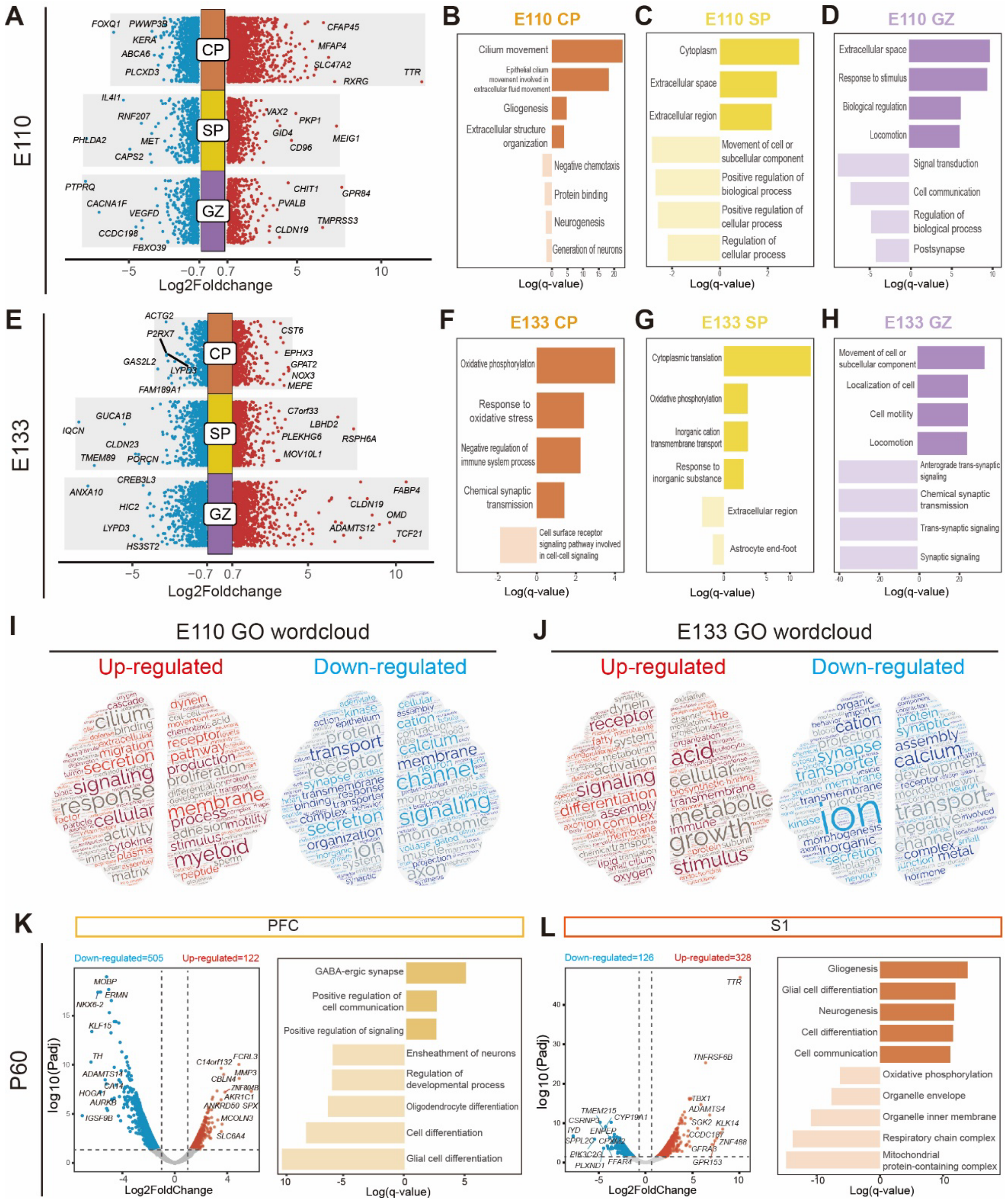

Figure S7. Transcriptome analysis of cortical laminae at E110, E133 and regional analysis of P60. (A) Volcano plot of E110 showing DEGs of CP, SP and GZ (|log2FC|>0.7, qvalue<0.05); **(B-D)** GO enrichment of **(B)** CP, **(C)** SP, and **(D)** GZ at E110; **(E)** Volcano plot of E133 showing DEGs of CP, SP and GZ (|log2FC|>0.7, qvalue<0.05); **(F-H)** GO enrichment of **(F)** CP, **(G)** SP and **(H)** GZ at E133; **(I-J)** Wordcloud of GO terms of **(I)** E110 and **(J)** E133, size represents the words show up frequency; **(K)** Volcano plot (left) and GO enrichment analysis (right) of the PFC region at P60 (|log2FC|>1, qvalue<0.05); **(L)** Volcano plot (left) and GO enrichment analysis (right) of the S1 region at P60 (|log2FC|>1, qvalue<0.05).

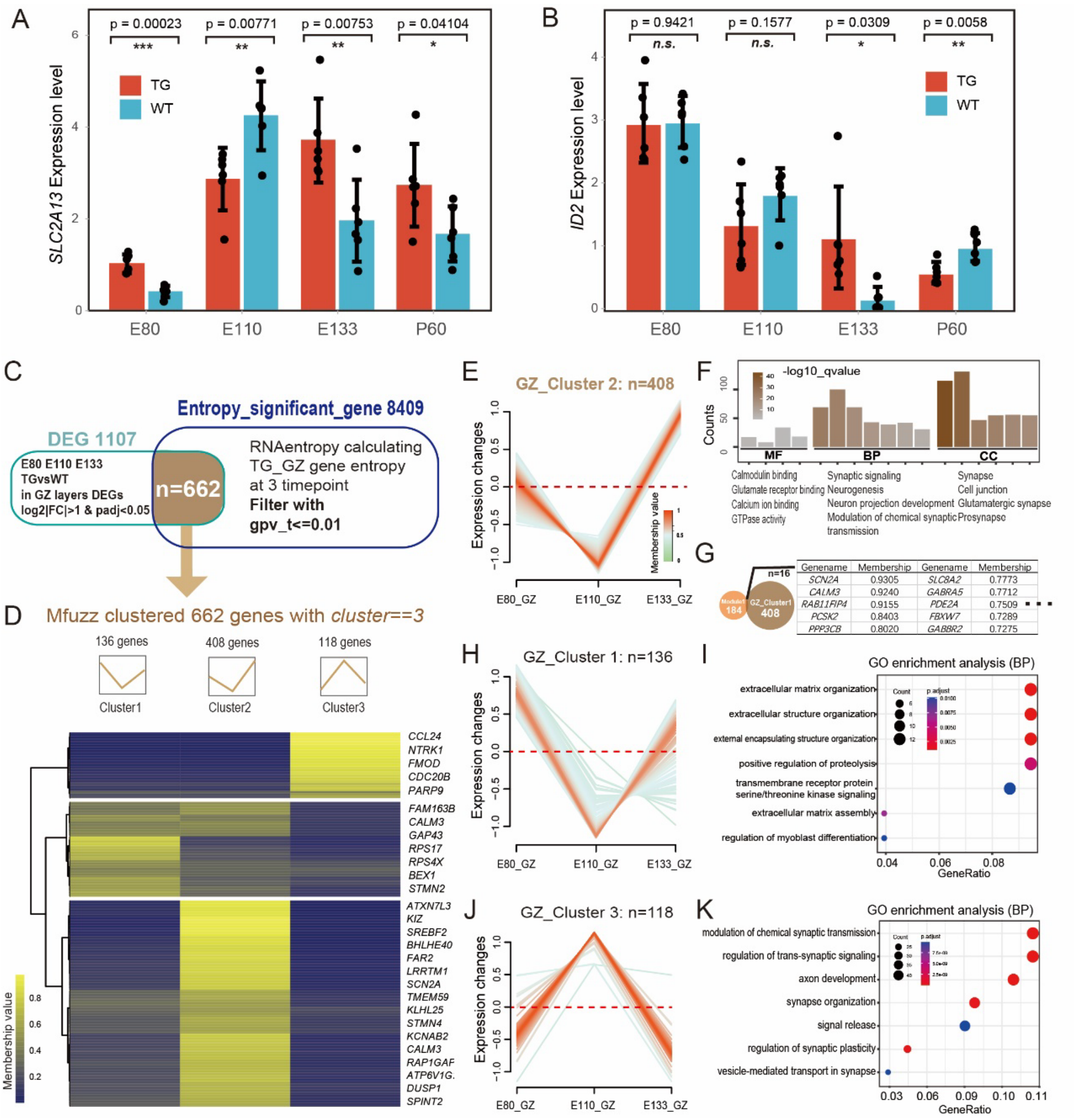

Figure S8. Patterns and functions of the GZ temporal DEGs. (A-B) Validation of **(A)** *SLC2A13* and **(B)** *ID2* expression level by qPCR (un-paired Students' t-test, * p<0.05, ** p<0.01, *** p<0.001, n.s. no significant); **(C)** The schematic of filtering temporal DEGs in GZ; **(D)** Heatmap of filtered GZ tDEGs; **(E-G)** tDEGs GZ-Cluster 2; **(E)** GZ-Cluster 2 genes expression changes trend graph; **(F)** GO enrichment of GZ Cluster2; **(G)** Intersection of Module1 and GZ Cluster2 genes; **(H-I)** tDEGs GZ-Cluster1; **(H)** GZ-Cluster 1 genes expression changes trend graph; **(I)** GO enrichment dot-plot of biological process; **(J-K)** tDEGs GZ-Cluster1; **(J)** GZ-Cluster 1 genes expression changes trend graph; **(K)** GO enrichment dot-plot of biological process;

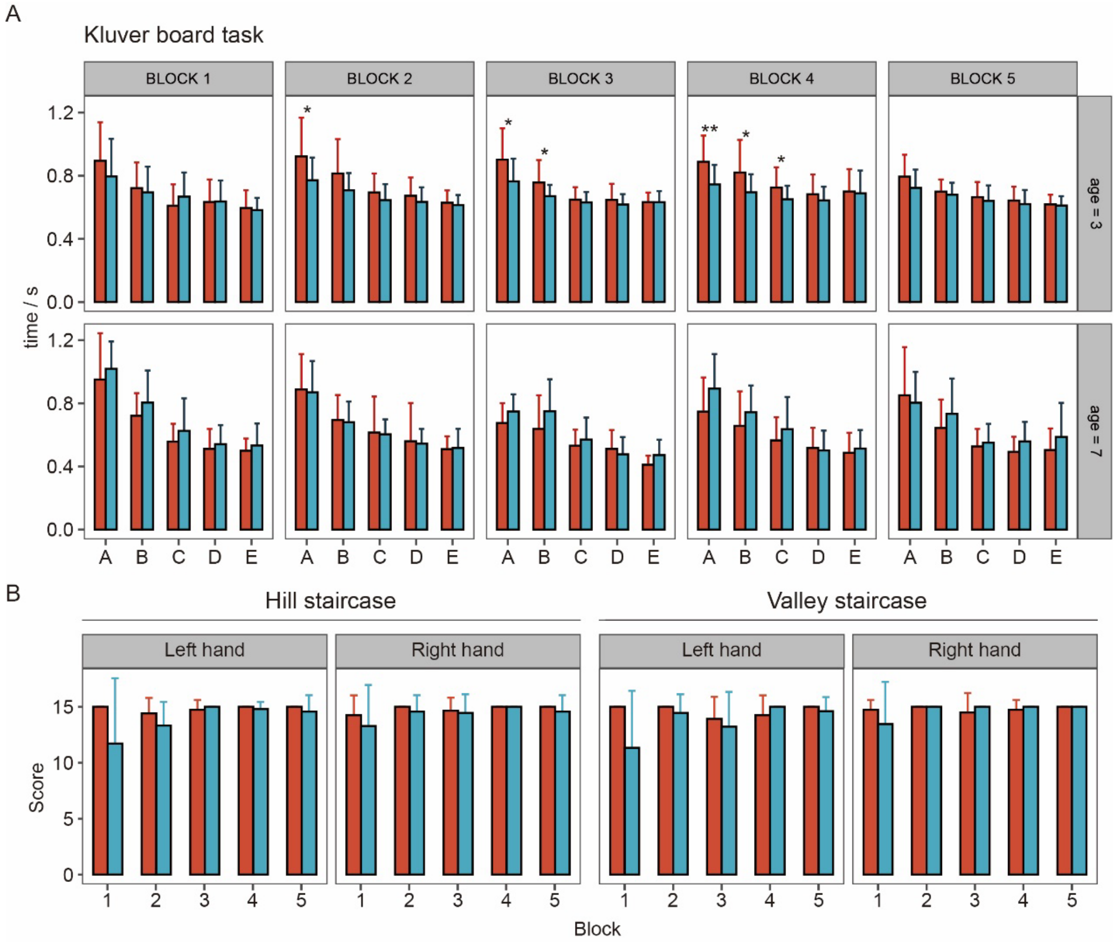

Figure S9. **The TG monkeys show better finger flexibility and complex motor planning and execution skills.** (**A**) The Kluver board task. Results are shown as time (mean ± standard error of the mean, the same below) in successfully retrieving food rewards from each hole during each block. Student's t- test, *, p<0.05, **, p<0.01. (**B**) The Hill-and-Valley Staircase tasks. Results are shown as score in successfully retrieving food rewards with the monkeys' left and right arms.

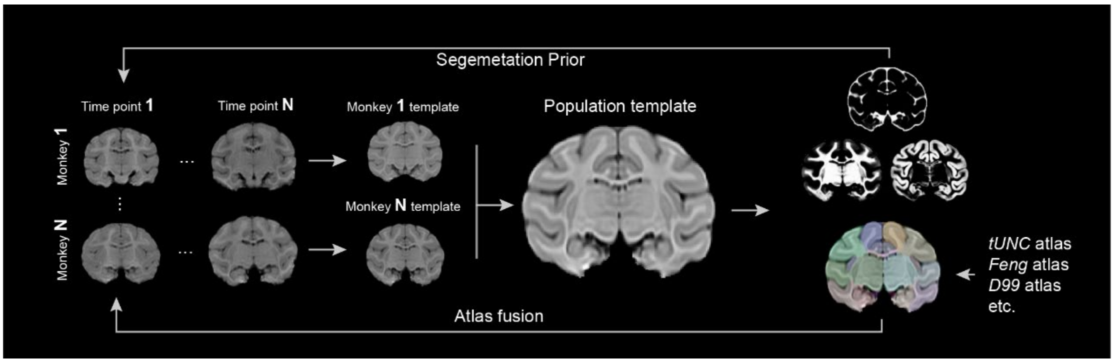

Figure S10. Schematic of T1-Weighted Template Construction. Following data preprocessing, individual templates were crafted using T1-weighted images across all time points for each monkey. These individual templates were then utilized to form a population template, from which population tissue probability maps were also generated. Brain parcellations from multiple macaque atlases were subsequently registered to this population template. Employing concatenated transformations obtained during template creation, these registered brain parcellations and population tissue maps were fused into individual T1-weighted images, serving as the foundation for future analyses.
