# Supplementary material for: Brain developmental and cortical connectivity changes in transgenic monkeys carrying the human-specific duplicated gene SRGAP2C
Source: Natl Sci Rev. 2023 Nov 3;10(11):nwad281. doi: 10.1093/nsr/nwad281 (PMC10712708; doi:10.1093/nsr/nwad281)
Supplement: nwad281_Supplemental_Files [file nwad281_supplemental_files.zip › Supplementary_Table_1.pdf]

Supplementary Table 1. Information of the wide-type monkeys.

Monkey ID	Generation	Sex	Status
WT01	F0	F	Sampled at Embryonic 78 days
WT02	F0	M	Sampled at Embryonic 78 days
WT03	F0	M	Sampled at Embryonic 78 days
WT04	F0	M	Sampled at Embryonic 78 days
WT05	F0	M	Live, birth at 2016/3/12
WT06	F0	F	Live, sampled at 3.7Y
WT07	F0	M	Live, birth at 2016/3/14
WT08	F0	M	Live, birth at 2016/3/22
WT09	F0	F	Live, birth at 2016/3/6
WT10	F0	F	Sampled at Embryonic 110 days
WT11	F0	M	Sampled at Embryonic 109 days
WT12	F0	M	Sampled at Embryonic 133 days
WT13	F0	F	Sampled at Embryonic 135 days
WT14	F0	F	Live, Sampled at 66 days after birth
WT15	F0	M	Live, Sampled at 60 days after birth
WT16	F0	M	Live, Sampled at 781 days after birth
WT17	F0	M	Live, Sampled at 782 days after birth
