# Supplementary material for: Brain developmental and cortical connectivity changes in transgenic monkeys carrying the human-specific duplicated gene SRGAP2C
Source: Natl Sci Rev. 2023 Nov 3;10(11):nwad281. doi: 10.1093/nsr/nwad281 (PMC10712708; doi:10.1093/nsr/nwad281)
Supplement: nwad281_Supplemental_Files [file nwad281_supplemental_files.zip › Supplementary Materials and Methods.docx]

**Materials and Methods**

**Animal ethics statement**

The use and care of animals were approved in advance by the Institutional Animal Care and Use Committee of Kunming Institute of Zoology, Chinese Academy of Sciences (Approval No: SMKX-2017021).

**Preparation of lentivirus**

Human *srGAP2C* coding region was amplified from FP655-pCAG-srGAP2C-EGFP with specific primers and cloned into backbone vector pLV-Fhsyn-2A-EGFP, and we obtained a reconstructed lentiviral vector pLV-Fhsyn-srGAP2C-2A-EGFP (PSSE). A total of 2.5 × 10^6^ 293T cells were seeded in a 10cm dish in priority. Transfer the culture medium to 6ml OPTI-MEM medium (Gibco) before transfection. The packing plasmids PSSE, PsPAX2 PMD2.G were mixed in appropriate proportion (5:3:2) in OPTI-MEM medium with lipofectamine2000 (Invitrogen) and keep for 20 min at room temperature. Then transfer the mixture to the cultured cells. 8 hours later, change the culture medium to Dulbecco Modified Eagle Medium (Gibco) with 10% fetal bovine serum (Hyclone) and continue culturing for another 36 hours. The supernatant was harvested and simply centrifuged to remove the remained cell debris. Then the supernatant passed through a cellulose acetate filter (pore size, 0.8um), and concentrated by ultracentrifugation (25,000 RPM, 2h). The viral pellet was resuspended in PBS, frozen, and titrated by qPCR.

**Collection of rhesus monkey oocytes and gene delivery**

Cycling females (6 to 12 years old) were subject to follicular stimulation using twice-daily intramuscular injections of 18 IU of recombinant human FSH (rhFSH) (Gonal FTM Laboratories) for 8d; then 1,000 IU of human chorionic gonadotropin (hCG) (Lizhu Groups) were injected on day 9 as described by Niu et al. The oocyte was collected by laparoscopic follicular aspiration 30-34 h following hCG administration. The collected oocytes were cultured in the pre-equilibrated Hepes-buffered Tyrode’s albumin lactate pyruvate (TALP) medium containing 0.3% BSA.

Metaphase II arrested oocytes were selected for perivitelline space injection of lentiviruses (1×10^10^ viral genome (vg) per ml) and ICSI. After microinjection, the oocytes were cultured in the maturation medium at 37 °C (in 5% CO2) for about 1 h, until fertilization by ICSI. A single oocyte was fixed by the holding pipette and a single sperm was immobilized and aspirated with the tail first. The injection pipette was pushed through the zona pellucida and subsequently through the oolemma to release the spermatozoon. After ICSI, the oocytes were cultured in HECM-10 containing 10% FCS (HyClone, Logan, UT) to allow embryo development. Culture medium was replaced every other day.

**Selection of surrogate mothers and embryo transfer**

Female monkeys exhibiting normal menstrual cycles were selected as surrogates. The menstrual cycles were recorded daily and the tubal embryo transfer was performed at 0-3 days after ovulation. 4-cell to blastocyst embryos were selected for tubal transfer by laparoscopy.

**Genotyping and copy number and integration site validation**

For embryo genotyping, two pairs of specific primers of *srGAP2C* and EGFP were chosen for nest PCR. Briefly, primer pairs1 was used for pre-amplification. Set annealing temperature at 60℃, and run for 10 cycles. The production could be used as template in the second run with primer pairs2. Set annealing temperature at 65℃, and run for 30 cycles. Then the PCR products were tested with agarose gel electrophoresis. For monkey tissue genotyping, the DNA was extracted from the tissue, and the PCR was performed with primer pair2 with the annealing temperature set at 65℃ and run for 35 cycles. the production could be tested with agarose gel electrophoresis.

The copy number validation was performed using the commercial reagent kit (Lenti-X™ Integration Site Analysis Kit, 040413, TAKARA) based on the protocol of the previous study[18], which uses DNA walking technology to locate the insertion site by targeting the LTR sequence of the entire genome. For the sampled monkeys, we chose to extract DNA from brain tissue for copy number identification. For the live monkeys, we extracted DNA from the ear epidermis, which is also derived from the ectoderm during development for identification.

**Western Blotting**

Proteins from the monkey brain were homogenized in RIPA lysis buffer (50mM Tris-HCl, pH 7.4; 150mM NaCl; 1mM EDTA;1% Triton-100; 1mM Na3VO4) containing a cocktail of protease inhibitor (Sigma Chemical, St. Louis, MO). Extracted proteins (15-20μg) were separated by SDS-polyacrylamide gel electrophoresis and electrophoreticly transferred to a membrane incubated with anti-GFP monoclonal antibody mouse anti-GFP (ThermoFisher, MA5-15256), Immunoreactivity was detected with an enhanced chemiluminescence system (Pierce Protein) with colored ladder (Bio-rad, 1610374) as the molecular size standard.

**MRI Data Acquisition**

Each monkey was subjected to MRI scans using a Philips Achieva 3.0T TX scanner. For the sedation protocol, an initial dosage of ketamine hydrochloride (10mg/kg) was administered, followed by pentobarbital sodium (10mg/kg) to ensure immobilization. T1-weighted (T1w) images were captured with a 3D turbo field echo sequence, detailed with parameters as follows: time repetition (TR) = 14 ms; time echo (TE) = 7 ms; flip angle (FA) = 8°; number of average (NA) = 3; matrix size = 320 x 320; Field of view (FOV) = 120 x 120 mm; slice thickness = 0.5 mm; voxel resolution = 0.375 x 0.375 x 0.5 mm3; scan duration = 8.58 mins. DTI data were acquired using a two-dimensional echo-planar diffusion-weighted spin-echo sequence (TR = 5.26 s; TE = 88 ms; FA = 90°; NA = 4; matrix size = 160 x 160; FOV = 120 x 120 mm; slice thickness = 1.2 mm; voxel resolution = 0.75 × 0.75 × 1.2 mm3; b = 800 s/mm2; 32 directions with one non-diffusion weighted image; scan duration = 12.75 mins).

**T1-w data preprocessing**

The initial step in preprocessing involved the extraction of brain regions. This was accomplished by registering and fusing the infant macaque atlas to each subject's individual scan to produce an initial brain mask, which was then manually fine-tuned, similar as our previous studies [19][17]. Automated post-processing strategies were subsequently applied to minimize the subjective human errors that might arise from manual adjustments. FSL's FAST [65] algorithm was utilized to correct the bias field and segment the T1w images into gray matter, white matter, and cerebrospinal fluid compartments. Owing to the incomplete myelination in the white matter of infant monkeys, default k-means segmentation in FAST/FSL could lack precision. To enhance the segmentation quality, tissue probability maps derived from older time-points were employed as priors for re-segmenting the T1w images, thus effectively improving the anatomical delineation in the infant monkey brains. Cortical thickness was assessed using the DiReCT method as implemented in the ANTs software [66]. All of the T1w images were then aligned to a study-specific template using ANTs for further region-of-interest (ROI) analyses based on the infant macaque atlas.

**DTI data Preprocessing and template construction**

Initial preprocessing corrected for motion artifacts and eddy currents using Tortoise [67]. Brain extraction was executed in a manner similar to that described for T1w data, using a semi-automated, template-based approach. Thereafter, diffusion tensor-based metrics such as fractional anisotropy (FA), mean diffusivity (MD), axial diffusivity (AD), and radial diffusivity (RD) were calculated. DRTAMAS was used to register all tensor images to a DTI template (ref.). The white matter ROIs from the tUNC atlas [68] and the Feng atlas [69] were mapped onto the DTI template.

**RNA isolation**

The tissue was sampled and quick-frozen in liquid nitrogen after monkeys were sacrificed within 24 hours. The RNA was extracted with a Qiagen RNEasy kit (74004). The RNA quality was checked by Agilent 2100.

**Laser microdissection and RNA isolation**

The fetal brain was firstly partitioned into several parts embedded in OCT and gradually frozen in a mixture of ethanol and dry ice. The frozen specimen contains prefrontal cortex (PFC) was cryosectioned at 30μm and the section was transferred onto polyethylene naphthalate (PEN) slides (Leica Microsystems) and placed the slides on dry ice immediately, and stored at -80°C until use.

Take the slides out from the -80°C freezer and balance at room temperature (RT) for 1 min. The slide was transfered into 95% EtOH for 30s, 75% EtOH for 30s, 50% EtOH for 30s, and DEPC H_2_O for 30s twice successively. Then the slide was transferred into Nissl staining solution and incubated for 40s at RT. When the staining was over, taking out the slide and passing into dehydration progress immediately. The dehydration reagent composed of (DEPC H_2_O twice, 50% EtOH, 75% EtOH, 95% EtOH, and twice pure EtOH) the slides stayed at most 10s. After air-drying, proceed with the slide immediately onto the laser capture microdissection (LCM, Leica LMD6000).

According to the cell density, the cell layers were determined under the microscope. The CP, SP, OSVZ, SVZ, and VZ were collected directly into a 1.5ml tube containing QIAzol buffer from the miRNeasy Mini Kit (217004, Qiagen). RNA was isolated following the manufacturer’s directions for the miRNeasy Mini Kit. RNA samples were run on the Agilent 2100 Bioanalyzer (Agilent Technologies) for quality testing.

**cDNA library preparation and RNA-seq**

The cDNA libraries for paired-end sequencing were performed with Total RNA-Seq Kit v2.0 (Life Technologies) according to the manufacturer’s instructions. The cDNA libraries were then sent to BGI (Shenzhen, China) for sequencing.

**Transcriptome analysis**

The raw data underwent stringent filtering to eliminate low-quality data prior to subsequent analysis. Specifically, we applied the following criteria: low-quality regions at the end of sequences were removed using the BWA algorithm with a threshold of 30. Sequences containing joint sequences and ambiguous base 'N' were trimmed from the raw data. Transcripts from each sample with read lengths < 60bp were excluded. These criteria resulted in the generation of high-quality, clean data suitable for further analysis.

We sourced the reference monkey genome and annotation files from the ENSEMBL database (http://www.ensembl.org/index.html). The cleaned data were aligned to the reference genome using HISAT2 (v2.0.5) (http://ccb.jhu.edu/software/hisat2/index.shtml), generating BAM files that contained read position information on the reference genome as well as sample-specific characteristics. Subsequently, we utilized the featureCounts software (http://subread.sourceforge.net/) to calculate read counts and Transcripts Per Million (TPM) for each sample, representing their respective expression levels.

Differential expression analysis was carried out using edgeR (https://bioconductor.org/packages/release/bioc/html/edgeR.html), followed by correction for multiple hypothesis testing using the Benjamini-Hochberg method. A common significance criterion was applied: adjusted p-value < 0.05 and |log2foldchange| > 0.7 for mRNA expression.

To evaluate functional enrichment, we conducted analyses of Gene Ontology (GO) Biological Processes and KEGG (Kyoto Encyclopedia of Genes and Genomes) pathways, considering them essential. For the differentially expressed genes (DEGs), we employed metascape (https://metascape.org/gp/index.html) and clusterProfiler (https://bioconductor.org/packages/release/bioc/html/clusterProfiler.html) for GO and KEGG pathway category analysis. Significantly enriched GO and KEGG categories were identified using a chi-square test with an adjusted p-value cutoff of 0.05. Multiple test corrections were applied using the Benjamini-Hochberg method.

Regarding the selection of genes for longitudinal analysis, we first filtered out genes with TPM values < 1 in each of the four stages (CP layer) and three stages (GZ layer) in subsequent analyses. To identify dynamic genes, we employed the RNentropy[42] package to calculate gene entropy, using a threshold of gpv_t < 0.01 in the TG group. This resulted in the dataset named TG "Entropy_significant_gene" representing genes that exhibit significant variability across epochs. Subsequently, we merged DEGs at each epoch using the same criteria (|log2FC| > 1 & padj < 0.05) between TG monkeys and WT control groups. Finally, we overlapped these two sets to obtain a dataset of temporal DEGs (tDEGs), representing genes that exhibited differential expression between groups and displayed significant trends across different epochs.

Subsequently, we employed RNentropy-calculated entropy values to perform clustering of tDEGs using Mfuzz [43]. The number of clusters was determined based on the number of time points ± 1, ensuring high convergence of gene expression curves within each cluster and representative gene trends. Following this criterion, for CP-tDEGs, clusters were successively reduced starting from *cluster =5*, while for GZ-tDEGs, the reduction began from *cluster =4.* Ultimately, 5 clusters were selected for CP and 3 clusters for GZ, respectively.

**Dye microinjection and morphology analysis of pyramidal neurons**

The animals were first sedated with ketamine hydrochloride and then euthanized using an overdose of sodium pentobarbital. Subsequently, they underwent intracardial perfusion with a solution composed of 0.1 M potassium phosphate-buffered saline at pH 7.2, followed by 4% paraformaldehyde in 0.1 M PB.

Dye microinjection was based on previous study [70][71]. Briefly speaking, after perfusion with a 4% PFA solution (0.01M PBS) and subsequent 12-hour fixation, brain tissue samples were carefully dissected to isolate regions of interest. Using a vibrating microtome (Leica, VT1200s), brain tissue slices with a thickness of 220μm were obtained. Glass electrodes with an outer diameter of 1.00mm and an inner diameter of 0.5mm (Sutter) were pulled to a tapered injector shape with a tip resistance of approximately 80-120mΩ using a micropipette puller (Sutter P-97). Subsequently, a dye solution (Alexa Fluor 568 Carboxylic Acid, ThremoFisher, A33081) was slowly introduced from the back end of the glass pipette, adding a total volume of 10μl.

The glass electrode was securely mounted on a holder and carefully adjusted to ensure that the tip was in the same plane as the tissue block. The microscope was then set to a magnification of 40×.Under CCD observation, pyramidal neurons located in the layer 2/3 of the cerebral cortex were targeted. The electrode tip was gently inserted into the cell until an approximate fluorescence cell morphology was visible under the fluorescence channel. The current-clamp amplifier was then activated, and a current of -5nA was applied for 8-9 minutes. Dendrites of the cell and details of dendritic spines gradually became evident. Subsequently, the current-clamp amplifier was turned off.

In Sholl analysis, morphologically intact pyramidal neurons located in layer 2/3 of the cerebral cortex were selected, ensuring a clean background and complete apical dendrites. Utilizing a confocal microscope with a 10x objective lens, three-dimensional images of the entire cell morphology were captured along the z-axis. Subsequently, ImageJ software was employed to extract cellular morphological features, facilitating the completion of concentric Sholl analysis [71].

Dendritic spine analysis encompassed density statistics and density analysis following spine classification. Using a 63x confocal microscope, fully extended apical dendrites were imaged along the z-axis, and spine density was quantified per unit length. Spines were classified into four major categories based on traditional criteria: 1) Mushroom: Spines with a head diameter (Dh) ≥ 1.5 times the neck diameter (Dn) (Dh/Dn ≥ 1.5); 2) Stubby: Spines with head and neck diameters in close proximity, and spine length (L) not significantly longer than head diameter (Dh/Dn < 1.5, L/Dh < 2); 3) Thin: Spines with consistent head and neck lengths, and spine length noticeably longer than head diameter (Dh/Dn < 1.5, L/Dh ≥ 2); 4) Filopodia: Spines resembling Thin spines but lacking a distinct head and with greater length (Dh/Dn < 1, L/Dh ≥ 4).

**Immunofluorescent staining**

Briefly, embryonic monkey brains (E80, E110, E133) were dissected immediately and washed with pre-cooling PBS (Gibco). Postnatal monkey brains (P60) were fixed by sequential intracardial perfusion with saline at room temperature (RT), and 2Y monkeys needed to be perfused with 4% PFA in PB as a dye microinjection requirement. The brain tissue was embedded in Tissue-Tek (Sakura Finetek) and gradually cooled using dry ice dissolved in ethanol to reach -80°C. Cryosections with a thickness of 20 µm were prepared and stored at -20°C until needed. Subsequently, the cryosections underwent a 30-minute incubation at 70°C in an antigen retrieval solution (10 mM sodium citrate buffer, pH 7.0). After being balanced to room temperature, the sections were permeabilized using 0.5% Triton X-100 in 0.1M phosphate-buffered saline (PBS) for 30 minutes. Quenching of PFA was achieved with 0.1M glycine in 0.1M PBS for another 30 minutes, followed by blocking with 5% Bovine Serum Albumin (BSA) at room temperature for 30 minutes. Primary antibodies were applied to the brain slices overnight in the blocking solution, followed by appropriate fluorophore-conjugated secondary antibodies. Finally, the sections were counterstained with DAPI.

Primary antibodies: rabbit anti-Satb2 (abcam, ab92446, 1:1000), rat anti-Ctip2 (abcam, ab18465, 1:500), mouse anti-GFP (ThermoFisher, MA5-15256, 1:500), rabbit anti-Ki67 (abcam, ab16667,1:500), mouse anti-Nkx2.2 (DSHB, 74.5A5, 1:200), rabbit anti-Olig2 (abcam, ab109186, 1:500).

Secondary antibodies: AlexaFluor 488 goat anti-rabbit IgG (abcam, ab150077, 1:1000), AlexaFluor 488 donkey anti-mouse IgG (abcam, ab150105, 1:1000), AlexaFluor 594 goat anti-rat IgG (abcam, ab150160, 1:1000), AlexaFluor 647 goat anti-rabbit IgG (abcam, ab150083, 1:1000)

Images were acquired on Leica SP5 confocal microscope system. Quantification and analysis were carried out using Fiji (https://imagej.net/software/fiji/) software.

**General behavioral observation**

A total of 8 monkeys including 4 wild type and 4 *srGAP2C* transgenic monkeys were included in the general behavioral observation and cognitive test. All procedures were under the guidance of IACUC of Kunming Primate Research Center, Kunming Institute of Zoology, Chinese Academy of Science (Approval No: IACUC19003).

We used focus animal sampling to collect general daily behaviors [72]. For five consecutive days, each monkey was monitored for an hour daily, starting from 2 p.m. to 3 p.m. To ensure the monkeys were accustomed to the monitor's presence, it was placed three days prior to the recording. Recordings that were pieced together and labeled with letters were evaluated by three separate viewers. Classification and definitions of behaviors were based on previous studies [17][50][51]. Briefly, self-injuring behavior including self-biting and self-hair plucking were defined as severe abnormal behavior, repetitive and consistent actions were termed as stereotypical behaviors, including licking/biting bars, rubbing bars, stereotypic pacing/flipping, hand stereotype, saluting/eye poking, self-clasping, and floating limb, and other normal daily behaviors include locomotion, self-grooming and resting.

The same approach was used to examine the sleep patterns of monkeys. The video system is composed of two infrared cameras that point toward the cages and a hard disk video recorder that stores the recordings. Video recording equipment was placed three days in advance to familiarize the monkeys. Each monkey was videotaped from 18:30 to 8:30 for four consecutive nights. The mosaicked videos were analyzed by three viewers who were kept in the dark about the monkeys' information. Previous ethology studies on macaques and baboons have outlined the sampling method [17][52]. Monkeys presented 1-2 times body or limb movement within 1 min and were considered transitional sleep. Monkeys maintained a sitting or lying position, with the head always bent below the shoulders or backward, and with no body and limb movements, were considered to have relaxed sleep. Both conditions were recorded as sleep states. Monkeys were considered awake only when they showed locomotion occurred or over 3 times body movement within 1 min. Awake and sleep were recorded in 1-minute epochs. Those less than one minute are rounded off.

**Manual dexterity test**

We selected the Kluver board for the evaluation of hand flexibility [73], which is a transparent acrylic board with 5 circular holes with a diameter of 10mm to 13mm and a depth of 7mm. During each training session, the Kluver board was placed in front of the monkey cage, and a digital video camera was positioned to capture the board and monkey cage locations. Food was inserted into one of the holes using forceps, and the time required for the monkey to retrieve the reward was analyzed through video playback slowdown. We tested the monkeys at two time periods, including 3-4 and 6-7 years of age.

**Hill and Valley Staircase tasks**

We employed the device often used in marmosets to perform the hill and valley staircase tasks in cynomolgus monkeys [53][54]. Briefly, to receive food rewards from the steps of two staircases located outside the cage, monkeys are required to insert their arms through the vertical slots of a Plexiglas screen attached to the front of the cage. On each stair of the staircases, a peanut pellet is situated, totaling five pieces on either side. The monkeys were allowed 2 min to retrieve all the food bits, and this was recorded by a video camera and analyzed. Only successful reaches, defined as food taken securely through the slot, are counted. The score for each piece depends on the distance from the relevant slot (score 1 for the nearest piece, 5 for the farthest piece). The total score is summed to give a maximum score of 15 for each side. We also calculated the cumulative time required to pick up the food pellet. Food pellets that were not taken within two minutes were recorded as being taken for 120s.

**Statistical analyses of the MRI data**

For the analysis of MRI data, firstly, the normality of the data was assessed using the *Shapiro.test()* function in R. Then, we assessed the impact of age, group membership, and age-by-group interactions on volumes, as well as on fractional anisotropy (FA) and mean diffusivity (MD). We employed a linear mixed model represented by the equation: Y (T1-based volume or DTI value) = Intercept + A * age + B * group + C * age × group + random error (1|weight). The selection of this model involved an initial identification of the most representative variables through a subset regression process, followed by a comparison of Akaike Information Criterion (AIC) values to identify the optimal model. Subsequently, we conducted an ANCOVA F-test. To account for multiple tests, p-values for the group effect were adjusted using the Bonferroni correction. Our analysis was conducted using R version 4.2 (https://www.r-project.org/) and R Studio (https://www.rstudio.com/).

The pictures were obtained using R version 4.2, while the wordcloud was generated through an online platform (https://www.wordclouds.com/), and the animal icons were downloaded from the website (<https://www.biorender.com/>).

**References**

65. Smith SM, Jenkinson M, Woolrich MW *et al.* Advances in functional and structural MR image analysis and implementation as FSL. *Neuroimage* 2004;**23 Suppl 1**:S208-19.

66. Avants BB, Tustison NJ, Song G *et al.* A reproducible evaluation of ANTs similarity metric performance in brain image registration. *Neuroimage* 2011;**54**:2033–44.

67. Pierpaoli C, Walker L. TORTOISE: an integrated software package for processing of diffusion MRI data. *ISMRM 18th annual meeting* 2010;**51**:2010.

68. Irfanoglu MO, Nayak A, Jenkins J *et al.* DR-TAMAS: Diffeomorphic Registration for Tensor Accurate Alignment of Anatomical Structures. *Neuroimage* 2016;**132**:439–54.

69. Zakszewski E, Adluru N, Tromp DPM et al. A Diffusion-Tensor-Based White Matter Atlas for Rhesus Macaques. *PLoS One* 2014;**9**:e107398.

70. Dumitriu D, Rodriguez A, Morrison JH. High-throughput, detailed, cell-specific neuroanatomy of dendritic spines using microinjection and confocal microscopy. *Nat Protoc* 2011;**6**:1391–411.

71. Cao H, Li MY, Li G *et al.* Retinoid X Receptor α Regulates DHA-Dependent Spinogenesis and Functional Synapse Formation In Vivo. *Cell Rep* 2020;**31**:107649.

72. Altmann J. Observational study of behavior: sampling methods. *Behaviour* 1974;**49**:227–67.

73. Sugiyama Y, Higo N, Yoshino-Saito K *et al.* Effects of early versus late rehabilitative training on manual dexterity after corticospinal tract lesion in macaque monkeys. *J Neurophysiol* 2013;**109**:2853–65.
